# Natural‐Based Nanocomposite Ink Engineering for Seamless Multi‐Material Integration in Extrusion‐Based 3D Printing

**DOI:** 10.1002/adhm.202502733

**Published:** 2025-09-06

**Authors:** João R. Maia, Miguel Bilo, Daniel S. Fidalgo, Pedro D. Rebolo, Ana S. Silva, Marco Parente, Rita Sobreiro‐Almeida, João F. Mano

**Affiliations:** ^1^ Department of Chemistry CICECO – Aveiro Institute of Materials University of Aveiro Aveiro 3810‐193 Portugal; ^2^ Institute of Science and Innovation in Mechanical and Industrial Engineering (INEGI) R. Dr. Roberto Frias 400 Porto 4200‐465 Portugal

**Keywords:** 3D printing, bioactive glass, finite elements, multi‐tissue, nanocomposites, natural‐based, tissue engineering

## Abstract

Multi‐tissue regeneration remains a critical clinical challenge due to the lack of solutions that can replicate the hierarchical heterogeneity of such complex interfaces. While biofabrication approaches, such as extrusion‐based, allow replicating robust, biomimetic, and layered designs, constructs are usually hindered by inadequate phase/layer integration, poor filler dispersion, and mismatched rheological and mechanical performances. This study introduces an ink engineering strategy as a solution for integrating natural‐based nanocomposites in multi‐tissue regenerative approaches. For that, two photocrosslinkable natural matrices: a protein–bovine serum albumin methacrylate (BSAMA), and a polysaccharide–hyaluronic acid methacrylate (HAMA)–are selected for their complementary mechanical and cytocompatibility profiles. Bioactive glass nanoparticles, known for osteoconductive potential, are functionalized and covalently immobilized within both matrices through EDC/NHS chemical coupling. This primary crosslinking enables uniform distribution of inorganic phases, unlocking tuneable rheological properties, adequate for extrusion 3D printing. Then, a secondary crosslinking, leveraging the photo‐responsive moieties for post‐printing photocuring, enables the obtention of seamlessly integrated robust multi‐material constructs. Overall, BSAMA‐based inks offer higher cytocompatibility, while HAMA‐based inks provide superior mechanical strength. Their combination in multi‐material constructs supports hASCs’ metabolic activity and proliferation, confirming bioactivity and cytocompatibility; and finite element modeling validates their mechanical performance, supporting clinical potential for multi‐tissue regeneration.

## Graphical abstract

This innovative ink engineering strategy enables the integration of bioactive nanocomposites into complex, multi‐material constructs. By combining primary chemical coupling and post‐printing photocuring, the study achieves tunable printability, seamless material integration, and robust mechanical performance. Experimental and computational validation reveal high cytocompatibility and highlight the clinical translational potential, paving the way for personalized, multi‐tissue therapeutic applications.

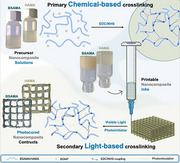

## 1 Introduction

Multi‐tissue regeneration remains a significant clinical challenge, with current treatments failing to ensure long‐term functional restoration.^[^ ^1^ ^]^ To effectively mimic native tissue, regenerative strategies must incorporate hierarchically arranged multilayered scaffolds with seamless interfaces between tissues of different natures and properties.^[^ ^2^, ^3^ ^]^ Additionally, bioactivity is crucial for inducing regeneration and facilitating phase integration, ensuring a mechanically robust and biologically active scaffold. However, current approaches lack ideal multilayered scaffolds that combine biomimetic architecture with adequate support for tissue regeneration.^[^ ^4^ ^]^

In tissue engineering (TE), composite materials comprising an organic biodegradable matrix and an inorganic resorbable filler are usually developed to acquire tailored properties that surpass those of individual components.^[^ ^5^ ^]^ Native bone consists of hydroxyapatite‐reinforced collagen, while cartilage is composed of highly organized collagen fibers. Most multi‐tissue TE strategies attempt to replicate such compositions by combining bioactive nanofillers with a supportive biomaterial matrix.^[^ ^6^, ^7^, ^8^ ^]^ Among these, bioactive glass nanoparticles (BGNP) are particularly promising due to their ability to promote both subchondral bone and cartilage regeneration.^[^ ^9^ ^]^ When exposed to physiological fluids, BGNPs bind ions and form bone‐like hydroxyapatite, greatly enhancing in situ regeneration.^[^ ^10^ ^]^ In this context, nanocomposite (NC), materials integrating nanostructured components are considered ideal for rendering additional bioactivity into scaffolds, as they effectively interact with biological structures at the nanoscale. The composition, concentration, and dispersion of nanofillers within the matrix strongly influences the mechanical and biological performance of the final construct.^[^ ^11^, ^12^, ^13^ ^]^ Furthermore, osteoconductive fillers, which also comprise BGNPs, play a key role in forming a robust interfacial bond between the scaffold and surrounding tissue. Hydrogel‐based composites, particularly those derived from natural polymers, have been shown to facilitate this necessary scaffold integration.^[^ ^14^ ^]^

3D printing is the most coveted methodology for generating architecturally complex biomimetic constructs, enabling recreation of the microarchitecture of native tissues. Hydrogel‐based scaffolds often suffer from poor cell–scaffold interactions, nutrient diffusion limitations, and inadequate oxygenation. These issues can be addressed using 3D printing by introducing controlled porosity into scaffold designs.^[^ ^15^, ^16^, ^17^ ^]^ On the other hand, the introduction of nano‐sized fillers in 3D printing presents unique challenges, such as poor dispersion, suboptimal rheological properties, and needle clogging, which hinder their application.^[^ ^18^ ^]^ Advances in ink and material engineering offer potential solutions to address these limitations by enhancing the mechanical and rheological properties of printable materials.^[^ ^19^ ^]^ The chemical crosslinking between functionalized fillers and the biomaterial matrix can enable uniform nanofiller dispersion while endowing the composite with tailorable rheological properties and improving the composite's mechanical integrity.^[^ ^20^, ^21^, ^22^, ^23^, ^24^ ^]^ This strategy can also permit the printability of otherwise unprintable formulations for extrusion 3D printing.^[^ ^19^ ^]^

Previous multi‐tissue regeneration strategies have typically relied on a hydrogel upper layer and a bioceramic/polymeric lower layer. However, these multi‐material constructs often fail due to the incompatibility of materials across multi‐tissue interfaces.^[^ ^25^ ^]^ Current consensus suggests that using a consistent matrix throughout multilayered scaffolds can facilitate the tissue interface reconstruction.^[^ ^26^, ^27^ ^]^ Natural biopolymers such as hyaluronic acid (HA) and bovine serum albumin (BSA) are widely used due to their cytocompatibility, but their poor mechanical and rheological properties remain a limitation. However, photocrosslinkable biomaterials, including hyaluronic acid methacrylate (HAMA) and bovine serum albumin methacrylate (BSAMA), are increasingly being incorporated into composite formulations for TE applications.^[^ ^28^, ^29^ ^]^ The introduction of methacrylate functional groups into the biomaterial matrix enables spatiotemporal control over crosslinking, making post‐printing photocuring a viable strategy for preserving scaffold structure.^[^ ^30^, ^31^ ^]^ This property can also be exploited to seamlessly integrate adjacent biomaterial matrices with similar reactive moieties, facilitating multilayered scaffold fabrication. Moreover, both matrices and derived NC inks and scaffolds possess different mechanical properties, which have been reported to guide cell differentiation toward either chondrogenic or osteogenic lineages.^[^ ^32^, ^33^ ^]^

Computational methods are an increasingly valuable tool for accelerating biomaterial development in a cost‐effective and efficient manner. Such simulations have already been applied to biomaterials and hydrogels in biomedical applications.^[^ ^34^ ^]^ When integrated with biomaterial design and scaffold fabrication, computational modelling can predict construct feasibility and optimize material properties before fabrication, accelerating the translation of TE solutions into clinical practice.^[^ ^35^ ^]^ The Finite Element method has been used to simulate the 3D printing process of hydrogel‐based inks and to predict mechanical behavior and deformation. Methodologies such as these help to predict performance and improve scaffold design and fabrication processes for TE. Simultaneously, they support parameter optimization and reduce the need for costly experimental frameworks.^[^ ^36^ ^]^

In this work, we seek the immobilization of BGNP in both BSAMA and HAMA matrices via a primary chemical crosslinking of the composing NC phases through EDC/NHS carbodiimide zero‐length coupling chemistry. We hypothesize that this ink engineering strategy endows low viscosity matrices with suitable properties for their 3D printability by generating interfacial interactions between the composite phases. Simultaneously, the homogeneous distribution of BGNP ought to augment the overall bioactivity whilst enhancing the crosslinked scaffolds’ mechanical properties. BSAMA and HAMA's tunable and distinct mechanical, rheological, and cytocompatibility properties can therefore be leveraged into the fabrication of multi‐material constructs with integrated phase transitions for multi‐tissue TE. The application of extrusion‐based 3D printing, combined with computational simulations, is sought to provide a pathway for the creation of hierarchically structured multi‐tissue TE constructs with clinically relevant functionality.

## 2 Results and Discussion

### 2.1 Ink Engineering Strategy and Material Characterization

#### 2.1.1 Ink Engineering Strategy

**Figure 1:**
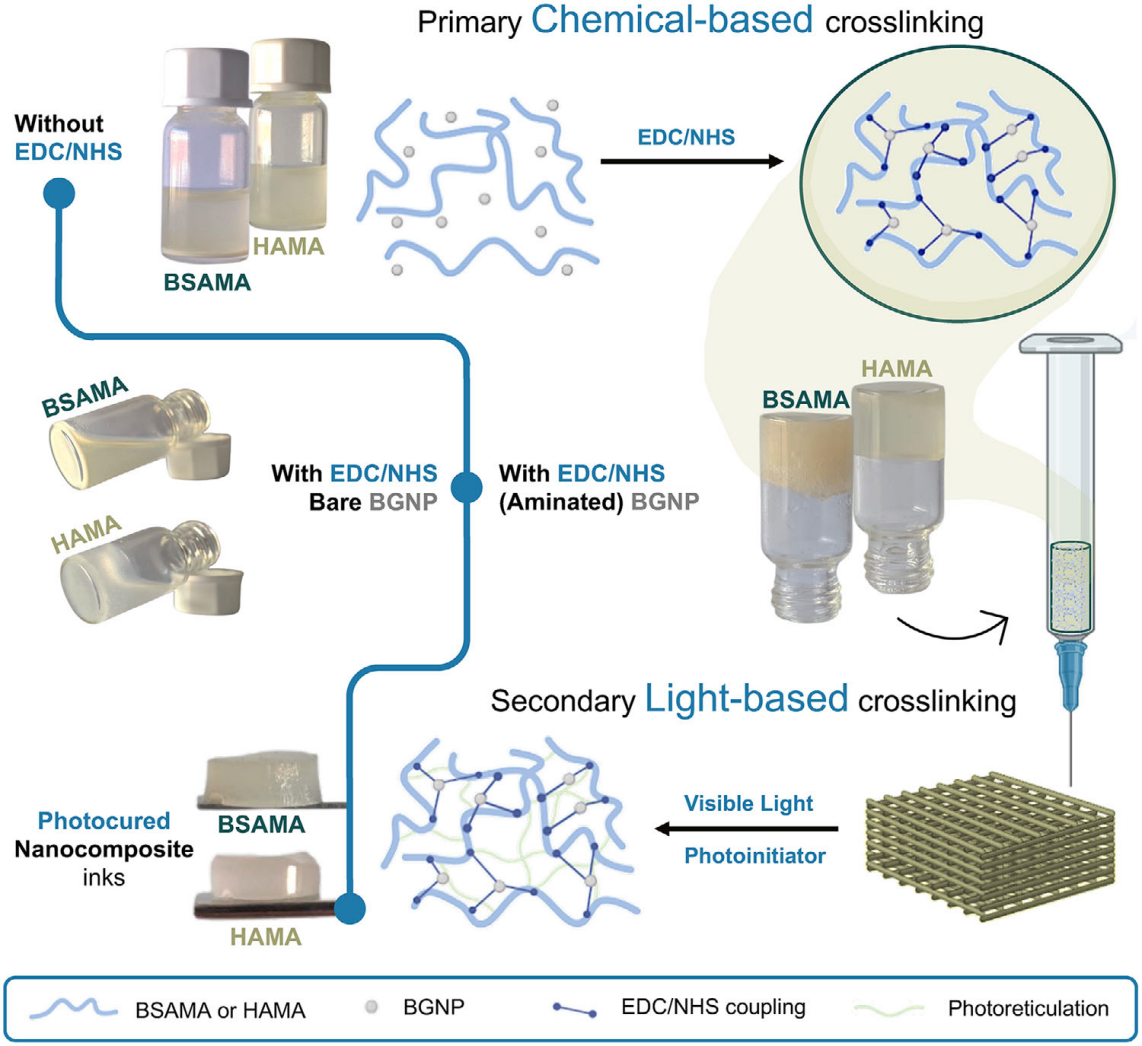
Ink engineering strategy for producing NC inks via a primary chemical crosslinking between the aminated BGNP and the matrices – BSAMA or HAMA. This primary crosslinking enables obtaining suitable properties for 3D printing. Because of their initial modification with methacrylate moieties, inks can also be post‐print photocured through a secondary light‐based crosslinking, for further mechanical reinforcement and maintenance of the printed structure.

Homogeneity of NC inks and their composing phases in combination with printable rheological properties are key features for extrusion 3D printing employment. The ink engineering strategy employed addresses these features simultaneously with a primary chemical‐based crosslinking strategy. This step endows homogeneity and printable properties by covalently crosslinking the NC's composing nanofiller and matrix phases. This crosslinking occurs via 1‐ethyl‐3‐(3‐dimethylaminopropyl) carbodiimide (EDC) and N‐hydroxysuccinimide (NHS) coupling chemistry, which binds carboxylic groups of BSAMA (10% w/v) or HAMA (5% w/v) with amine groups grafted to the BGNP. This type of two‐component EDC/NHS‐mediated crosslinking strategy has already proven its ability to provide mechanical reinforcement, phase integration, and even printable properties to composites or other protein matrices.^[^ ^19^, ^37^, ^38^ ^]^ These properties can also be further tuned per desired properties in terms of viscosity and moduli.^[^ ^19^ ^]^ This rationale is showcased in **Figure** **1**, which depicted first the NC phases mixture, which was liquid, unprintable, and showcased BGNP sedimentation, which could be reverted after the primary crosslinking took place, and became a homogeneous printable ink. The EDC/NHS amount was optimized to obtain adequate rheological properties on the NC inks. Also, because these properties are generated through the phase interaction, different BGNP amounts could be added. In this case, 5 and 10% w/w BGNP‐loaded formulations were studied. Further, by utilizing methacrylate‐modified matrices and photoinitiator – BSAMA or HAMA, and LAP, respectively – the NC gains spatiotemporal control over its final crosslinking ability.

The ^1^H‐NMR spectra of BSAMA and HAMA can be seen in Figure S1 (Supporting Information), which showcase their successful methacrylation. For BSAMA there are two peaks relating to the acrylic protons of the methacrylation (5.2 and 5.7 ppm range) that are highlighted, alongside a BSA backbone peak utilized to calculate the degree of modification (DoM).^[^ ^39^ ^]^ For HAMA, two peaks are highlighted at 1.8 and 1.9 ppm, corresponding to the methyl protons of the methacrylation and a backbone group of HA, respectively, which were used for the DoM calculation.^[^ ^40^ ^]^ BSAMA had a DoM of ≈80% and HAMA of 35%. These DoM were optimized according to the reactive groups available in each polymer to participate in the primary crosslinking using EDC/NHS. A high DoM in BSA was needed, given that the methacrylation takes place mainly in primary amines. In this way, protein–protein crosslinking is reduced, and protein‐nanoparticle bonds are optimized. In the case of HAMA, because no primary amines are available, only carboxylic acids participate in the primary crosslinking, as expected, which justifies the lower DoM.

This photocurable property was explored as a secondary light‐based crosslinking, as depicted in Figure 1, further enhancing the mechanical properties of the constructs. If introduced in a layered approach, it can effectively maintain the structure's shape and its integrity. Figure 1 also demonstrates that without the modification of the BGNP with amine groups, the ink engineering strategy cannot convey the same results (the pre‐crosslinking does not occur).

#### 2.1.2 Nanocomposite Phases’ Characterization

**Figure 2:**
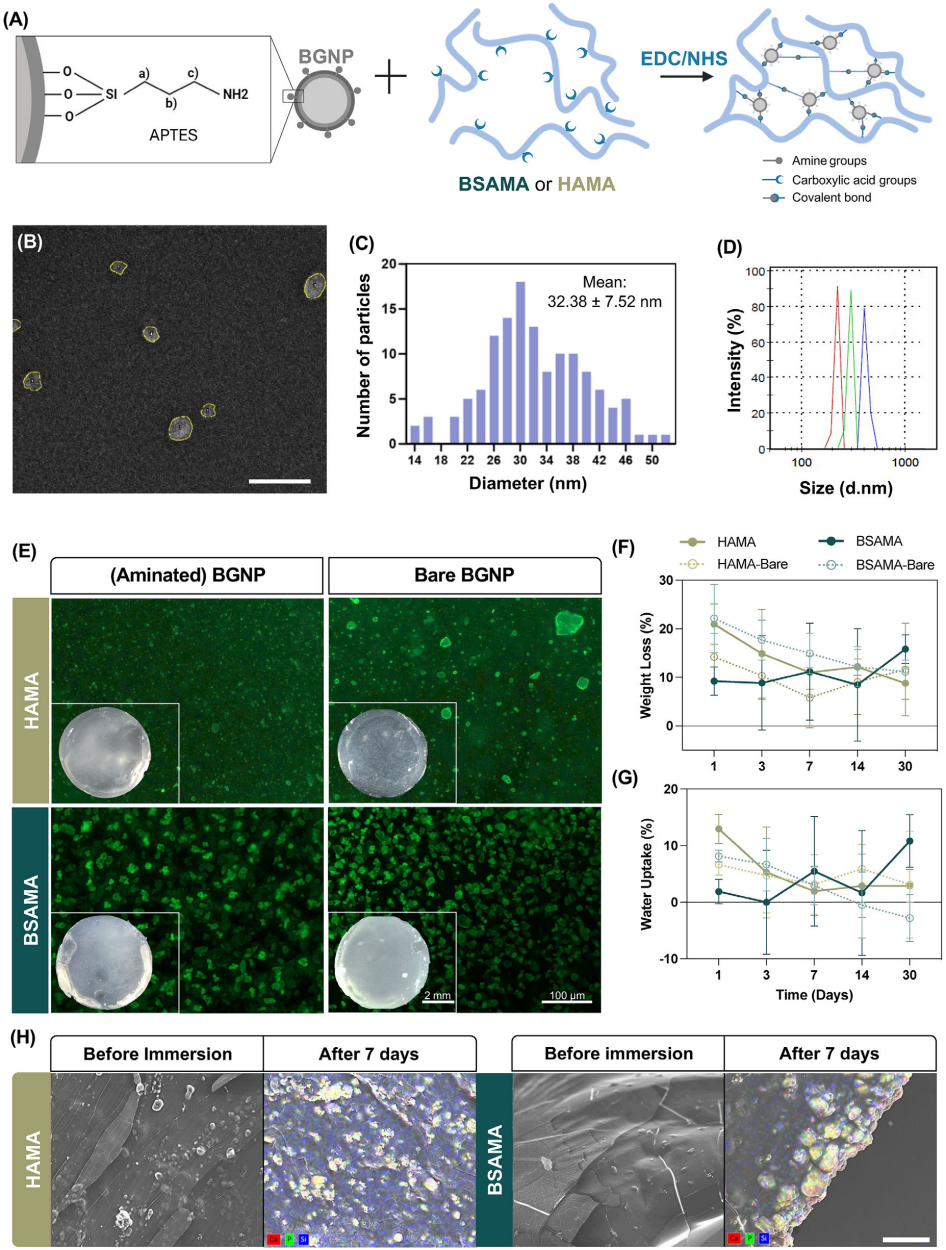
Material characterization: A) Schematic of BGNP surface modification with APTES for the primary chemical crosslinking with BSAMA or HAMA through EDC/NHS chemical coupling. B) TEM image of the BGNP. Scale bar: 100 nm. C) Particle size distribution, measured from TEM images using FIJI software.^[^ ^43^ ^]^ D) Particle size distribution acquired from dynamic light scattering. E) Osteoimage staining of aminated and bare BGNP NC scaffolds (10% w/w). F) Weight loss of 10% BGNP NC scaffolds for up to 30 days. G) Water uptake of 10% BGNP NC scaffolds for up to 30 days. H) SEM and SEM‐EDS images before and after 7 days of simulated body fluid assay on the left and right side, respectively. Scale bar: 100 µm.

**Figure** **2**A presents a schematic of the modification of BGNP with APTES, which, as previously reported, is necessary for the covalent bond and interface with the matrices, which is also represented. The BGNP mean particle size was 32.38 ± 7.28 nm, measured from transmission electron microscopy (TEM) micrographs (Figure 2B), which demonstrated their round shape and size assessment. The measured particle diameter counts are shown in Figure 2C. Additionally, Figure 2D displays the dynamic light scattering results, which accounted for the hydrodynamic radius with an ensemble average, with a mean of 254.25 ± 51.83 nm and confirming the nanometric scale in an aqueous dispersion.

In nanocomposites, it is well established that nanoparticles tend to agglomerate and sediment,^[^ ^41^ ^]^ which we aimed to mitigate by using covalent immobilization. Figure 2E showcases a staining for aminated BGNP and bare BGNP as a control in both HAMA and BSAMA scaffolds. The microscopy images showcased the different behaviors of the BGNP in BSAMA or HAMA NC formulations. Regarding aminated formulations, BSAMA had an increased particle agglomeration tendency in comparison to HAMA, which showcased a homogeneous distribution with minimal agglomeration. On the other hand, in the bare formulations, more agglomeration occurred in HAMA scaffolds, which can be related to its inherent high hydrophilicity. Furthermore, Figure 2E also showcases the scaffolds’ macroscopic evaluation. Especially for the case of HAMA scaffolds, the importance of the primary crosslinking was confirmed, given that when using bare BGNP, the scaffolds became noticeably heterogeneous. We have additionally performed SEM analysis to both bare and aminated NC scaffolds, in order to further corroborate this data and assess their inherent porosity (Figure S2A, Supporting Information). Our results reveal differences in surface porosity between BSAMA and HAMA aminated nanoparticle scaffolds (10% BGNP), the former showcasing higher surface porosity, in contrast with HAMA, revealing negligible surface porosity. Furthermore, when comparing the scaffolds of aminated versus bare BGNP, the latter displayed surface topography consistent with the presence of BGNP at the surface of the scaffolds. We hypothesize that this is the result of the lack of phase interaction, which in turn confirms the successful entrapment and immobilization of the nanoparticles in the aminated scaffolds.

To assess the behavior and stability of the NC scaffolds over time, weight loss and water uptake were performed for up to 30 days, comparing aminated and bare BGNP in NC scaffolds (Figure 2F,G, respectively). After 30 days, aminated BGNP BSAMA scaffolds showcased a weight loss of ≈15.8%, whereas HAMA of ≈8.8%. Scaffolds with bare BGNP had similar weight loss values after 30 days, of ≈11.2 and 11.6% for BSAMA and HAMA scaffolds, respectively. Regarding water uptake, after 30 days, aminated BGNP BSAMA scaffolds had a 10.8% increment, whereas their bare counterpart had a 2.8% decrease. For the HAMA scaffolds, the results between conditions were similar, where aminated BGNP HAMA scaffolds presented a 2.9% increment and 3.1% for the bare conditions. Both weight loss and water uptake results showcased no statistical differences throughout, either comparing within timepoints or formulations. By relating this data with the scaffold's SEM analysis (Figure S2A, Supporting Information), we consider that the samples should have negligible differences in porosity, which in turn does not affect water absorption rates. This lack of differences in behavior is most likely explained by the secondary crosslinking (photocuring), which, as previously stated, contributes more significantly to the overall characteristics of the NC scaffolds when compared with the primary crosslinking strategy. Although these inks and scaffolds may be lacking inherent porosity and pore interconnectivity, by employing extrusion 3D printing, nutrient and oxygen diffusion can be ensured by introducing micro‐to‐macroporosity during construct design.^[^ ^42^ ^]^

Regarding the bioactivity of the NC scaffolds, a simulated body fluid (SBF) immersion assay was performed for in vitro simulation of in vivo conditions. Figure 2H and Figure S2B (Supporting Information) represent SEM/EDS micrographs both after and before SBF immersion, respectively. For both HAMA and BSAMA scaffolds, there was a clear deposition of apatite‐like formations on the surface of the scaffolds. This is evidenced by both the elemental composition of these formations, as well as their characteristic “cauliflower”‐like morphology (Figure 2H). Before SBF immersion, BGNP can be observed on the surface of the scaffold but no evidence of apatite formation is seen, also corroborated by the absence of calcium and phosphate in these formations (Figure S2B, Supporting Information).

Overall, the primary crosslinking between the aminated BGNP and the organic matrices improved the homogeneous dispersion and immobilization of the particles in the bioactive scaffolds. Further, the secondary crosslinking strategy generated stable scaffolds that presented sustained degradation kinetics and water uptake capacity in similar rates. Both crosslinking strategies were paramount for achieving these properties, ensuring the best outcomes for the ink homogeneity and improved bioactivity, whilst forming a robust scaffold promising for osteochondral tissue implantation and regeneration.

### 2.2 Nanocomposite Inks’ Rheological Characterization

The rheological characterization of the NC formulations is imperative to assess the properties of the inks both before and after photocuring, and to predict their printability profile.^[^ ^44^ ^]^ To evaluate this, two NC formulations of different BGNP percentages were studied: 5 and 10% w/w. To compare the influence of particle inclusion, a control without BGNP addition was added (0% BGNP); in the same line of thought, to study the effect of the primary crosslinking, a control with 10% bare BGNP was also used.

The linear viscoelastic region was determined through strain sweeps (Figure S3A,B,E,F, Supporting Information) to be under 10% for all tested formulations. This value was applied in all subsequent rheological characterization testing. The NC formulations showcased a predominantly elastic behavior characterized by a prevalent elastic modulus (G′) compared to the viscous modulus (G″).

**Figure 3:**
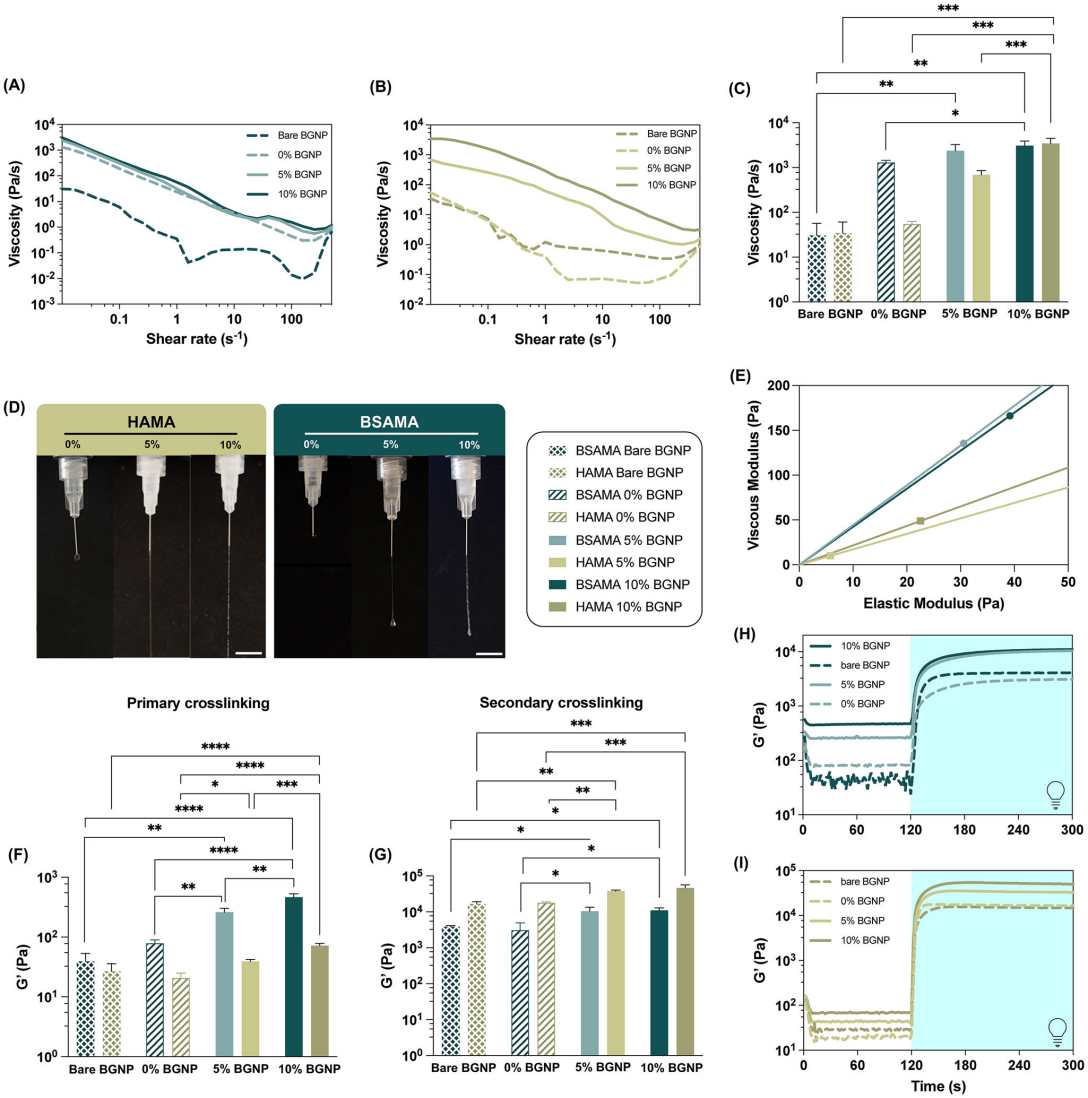
Rheological characterization (*n* = 3): A,B) Shear rate sweep for viscosity and shear‐thinning behavior assessment of BSAMA and HAMA NC inks and controls, respectively. C) Initial viscosity of BSAMA and HAMA NC inks and controls. D) Ink extrudability testing of NC inks with 0, 5, and 10% BGNP through a 27 G needle using a pump. Scale bars: 1 cm. E) Loss tangent values obtained from viscous and elastic moduli at 1% during strain sweeps for NC inks with 5 and 10% BGNP. F) Average storage moduli after the primary crosslinking for BSAMA and HAMA NC inks and controls. G) Average storage moduli after the secondary crosslinking for BSAMA and HAMA NC inks and controls. H,I) Photorheology time sweep with light exposure after 120 s of BSAMA and HAMA NC inks and controls, respectively.

To study whether the formulations could be employed in extrusion‐based 3D printing, their shear‐thinning behavior was tested through shear rate sweeps, which are showcased in **Figure** **3**A,B (Supporting Information) for HAMA‐ and BSAMA‐based formulations and controls, respectively. A shear‐thinning ink is imperative as it will decrease viscosity when experiencing higher shears, as happens when flowing through a nozzle/needle during printing. All formulations exhibited decreased viscosity with increased shear rate, which confirms their shear‐thinning properties. All control conditions revealed non‐linear behaviors, which also corroborates the necessity of the primary crosslinking to obtain homogeneous formulations. Shear rate sweeps, which evaluate viscosity, can also help to predict print fidelity. More viscous materials have increased print fidelity but entail higher shear stresses, which can hinder cell encapsulation capacity and post‐print elastic recovery.^[^ ^45^ ^]^ For the tested formulations, only the control's viscosity was not dictated by the primary crosslinking. This was confirmed in HAMA (Figure 3C), as the initial viscosity of the control was lower than for the NC formulations: ≈55 ± 8 Pa s^−1^ for 0% BGNP and 35 ± 27 Pa s^−1^ for bare BGNP. For the NC formulations, with increasing BGNP added, there was also an increment in the initial viscosity from ≈684 ± 176 Pa s^−1^ for 5% BGNP to 3443 ± 1092 Pa s^−1^ for 10% BGNP formulations. For the case of BSAMA formulations (Figure 3C), only the bare BGNP control had a lower initial viscosity compared to the remaining conditions (≈23 ± 26 Pa s^−1^). The rest of the formulations exhibited a similar tendency as HAMA, with increased initial viscosity with the addition of BGNP, from 1303 ± 154 Pa s^−1^ for the control without BGNP to 2379 ± 897 Pa s^−1^ with 5% BGNP added and 3082 ± 835 Pa s^−1^ for the 10% BGNP NC inks. This indicated that the presence of bare BGNP in BSAMA disturbs the system, impeding EDC/NHS crosslinking within the matrix itself. Yet, for BSAMA 0% BGNP, there was still a viscosity increment via the primary crosslinking due to the innate carboxylic and amine groups of BSAMA. In turn, this evidenced the primary crosslinking effect, where HAMA, which does not have these innate groups, had similar initial viscosity for both controls. In HAMA formulations, the NC phase interaction demonstrated having a larger effect on the acquired rheological properties, which was also in line with the results obtained in Figure 2E.

From the elastic and viscous moduli of the linear viscoelastic region of the NC formulations, the loss tangent (tan(δ)) can be calculated and plotted (Figure 3E). Tan(δ) can also advance if the ink has proper flow and/or shape retention, essential for 3D printing applications. Lower values are indicative of solid‐like behavior, and higher values of more liquid‐like behavior.^[^ ^45^, ^46^ ^]^ HAMA NC inks had similar tan(δ) values for 5 and 10% BGNP, of ≈0.58 and 0.46, respectively. For the case of BSAMA NC inks, tan(δ) values were also similar: ≈0.23 and 0.24 for 5 and 10% BGNP, respectively. The difference between HAMA and BSAMA and the similarity within formulations attested to the different nature of the inks, which is often the main influence of tan(δ). Further, compared to other reported printable inks, tan(δ) of the NC inks suggested their ability to be employed on extrusion 3D printing, with HAMA formulations being predictively more easily extrudable and printable, but entailing lower shape retention. This was also verified in the extrudability testing results presented in Figure 3D and Figure S4 (Supporting Information). Here, the ability of the NC inks to form cohesive filaments in a needle of up to 27 G was presented. This also confirmed the lack of printable properties of the control ink without added BGNP, which could not form filaments in either HAMA or BSAMA inks. Additionally, corroborating tan delta values, HAMA filaments demonstrated easy flowability, with higher filament length without breakage, when compared to BSAMA filaments.

Figure 3F,G presents the resulting G′ for all ink formulations and controls after the primary crosslinking strategy, as well as for the resulting scaffolds derived from the secondary crosslinking strategy. These results are a direct assessment from the photorheology time sweeps, presented in Figure 3H,I. The elastic modulus regarding the primary crosslinking was estimated before the photocuring of the ink (at 60 s) and for the secondary crosslinking after stability was reached (at 300 s). Regarding the primary crosslinking, 0, 5%, and 10% BGNP formulations exhibited the same pattern as for the acquired initial viscosity, with increasing values with the increase in BGNP addition. For HAMA, increased from 21 ± 3 Pa for 0%, to 39 ± 3 Pa for 5%, and to 72 ± 7 Pa for 10% BGNP added; for BSAMA, the increment was from 79 ± 11 Pa for 0%, to 261 ± 42 Pa for 5%, and to 465 ± 63 Pa for 10% BGNP added, revealing an almost linear correlation. For the controls with bare BGNP, their G′ was similar to the control without BGNP added, without statistical significance (27 ± 7 Pa for HAMA and 39 ± 14 Pa for BSAMA). The lack of statistical significance between controls, where the primary crosslinking cannot occur or is hampered, confirmed its influence on the acquired properties, such as an increment in G′. For both matrices, there was a statistical difference between adding 5 or 10% of BGNP, further showcasing the influence of the phase interaction. The acquired moduli of the NC ink formulations were comparable to previously reported values in similar works.^[^ ^18^, ^38^, ^47^, ^48^, ^49^ ^]^

Regarding the secondary crosslinking, the resulting G′ is a direct consequence of the photocuring of the formulations after the primary crosslinking has taken place. Here, for both matrices, there were no statistical differences between the 5 and 10% BGNP formulations. The only statistical differences were between these formulations and controls. For the case of HAMA, the statistical differences were more pronounced, with controls 5 and 10% BGNP NC scaffolds reaching 37.597 ± 2.206 and 46.4 ± 8.117 kPa, respectively. For BSAMA, 5 and 10% BGNP inks could respectively acquire 10.4 ± 3.116 and 10.971 ± 1.765 kPa G′ values. In Figure S3D,H (Supporting Information), we plotted the statistical significance between primary and secondary crosslinking within the same matrices. These results showcased that for both matrices, the acquired G′ of the NC inks are mostly impacted by the secondary light‐based crosslinking. When compared, HAMA and BSAMA resulting scaffolds showcased a clear disparity in the increment of G′ from primary to secondary crosslinking. HAMA NC scaffolds showcased approximately fourfold in G′ when compared to BSAMA NC. Overall, these results showed that for the final scaffolds, the photocuring (secondary crosslinking) had a larger impact on scaffolds’ properties than the primary crosslinking, corroborating previous results.

Figure S3C,G (Supporting Information) presents the results of frequency sweeps done on the photocured NC formulations for both 5 and 10% BGNP NC inks of HAMA and BSAMA, respectively. From these results, we could see that all tested formulations were stable up to 10 Hz. BSAMA NC scaffolds demonstrated a more frequency‐dependent behavior when compared to HAMA NC scaffolds, which could be explained by the larger contribution of the viscous component in these matrices. These results indicated that the NC ink formulations below this frequency have the expected behavior of solid‐like materials.

Overall, the rheological characterization and extrudability testing of the inks permitted a thorough characterization of the effect of the crosslinking strategies. The acquired viscosity was dictated by the primary crosslinking, which, accompanied by shear‐thinning properties, allowed the NC inks’ extrudability with proper flow and stable filament formation. BSAMA and HAMA inks demonstrated significant differences, not only in the final photocured scaffolds but also in the rheological properties of the ink, with HAMA conditions predictively giving enhanced flowability and uniformity for 3D extrusion printing. The secondary crosslinking allowed for the inks to further harden, increasing their modulus, with spatiotemporal control.

### 2.3 Mechanical Characterization of Nanocomposite Scaffolds

#### 2.3.1 HAMA and BSAMA Nanocomposite Scaffolds

**Figure 4:**
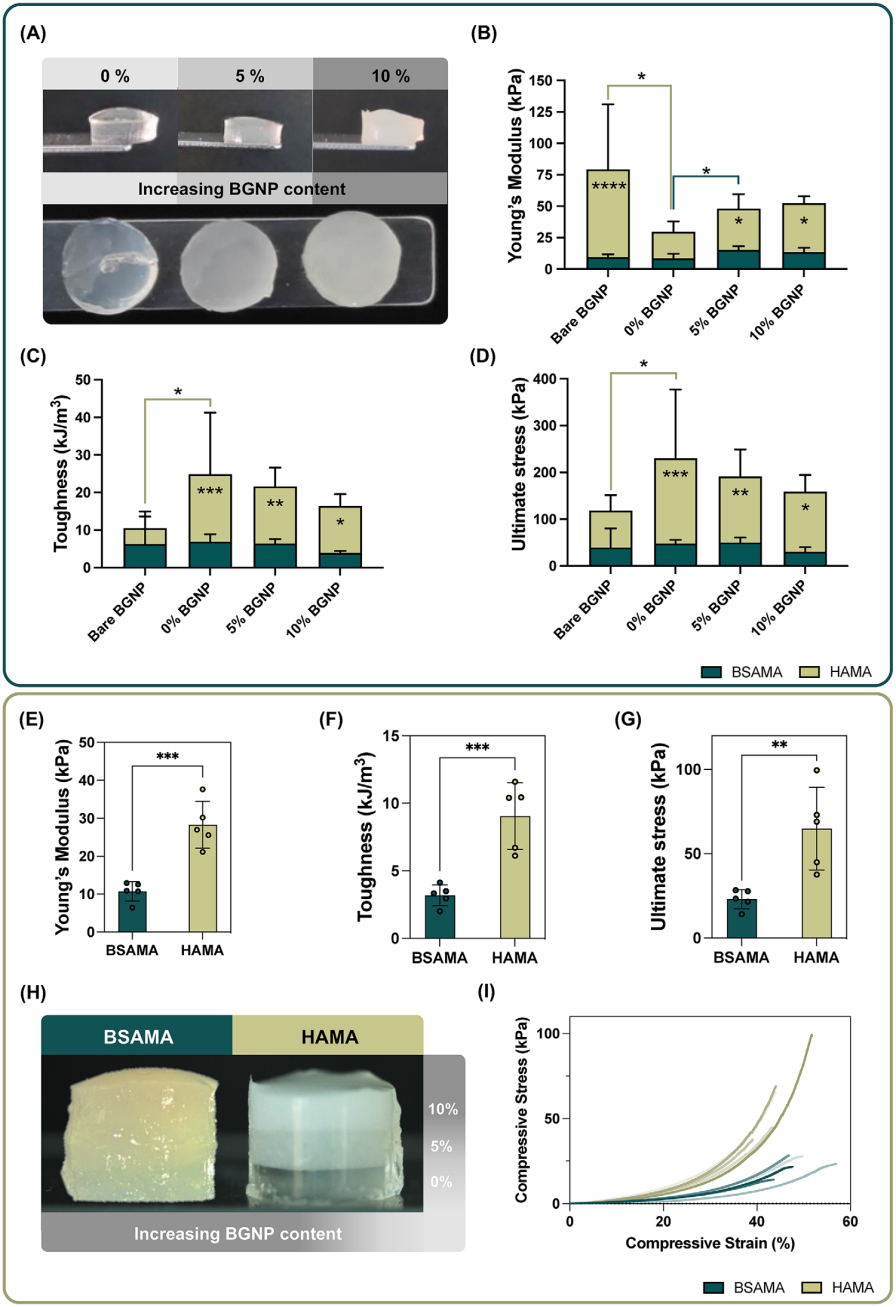
NC scaffolds and BGNP gradient scaffolds mechanical characterization: A,C,D) relate to the mechanical characterization of BSAMA and HAMA NC scaffolds containing 0, 5, and 10% of BGNP and 10% of bare BGNP (*n* = 5). E,F,G,I) relate to the mechanical characterization of BSAMA‐ and HAMA‐based BGNP gradient constructs (*n* = 5). A) Macroscopic pictures of HAMA NC scaffolds with 0, 5, and 10% BGNP. B) Young's Modulus of the HAMA and BSAMA NC scaffolds and controls. C) Toughness of the HAMA and BSAMA NC scaffolds and controls. D) Ultimate stress of the HAMA and BSAMA NC scaffolds and controls. E) Young's Modulus of the BGNP gradient HAMA and BSAMA constructs. F) Toughness of the BGNP gradient HAMA and BSAMA constructs. G) Ultimate stress of the BGNP gradient HAMA and BSAMA constructs. H) Microscopic pictures of the BGNP gradient HAMA and BSAMA constructs. I) Stress/strain replicate curves of the BGNP gradient HAMA and BSAMA constructs.

After assessing the rheological properties of the inks, it was paramount to evaluate the mechanical performance of the photocured scaffolds. Herein, the same formulations were tested for their response to external compressive force. This allowed assessing the influence of the BGNP addition and primary crosslinking on the mechanical properties. The inks and controls were pipetted after the chemical crosslinking and photocured in cylindrical molds for testing. **Figure** **4**A highlights examples of HAMA scaffolds tested. Figure 4B–D and Figure S5A (Supporting Information) present the results of the Young's modulus, toughness, ultimate stress, and ultimate strain values acquired from the compressive tests performed, respectively. Statistical inferences regarding comparison within the matrices are represented with lines of respective color, and differences between HAMA and BSAMA formulations are represented within the bar for each condition.

Young's modulus was calculated from the stress/strain curves of the compressive testing, presented in Figure 4B. HAMA NC scaffolds had the highest values with 48 ± 12 and 52 ± 6 kPa for 5 and 10% BGNP formulations, respectively. BSAMA NC scaffolds presented lower modulus with 15 ± 3 and 14 ± 3 kPa for the same BGNP percentages. 5% BGNP HAMA scaffolds had a 3.2‐fold increase in Young's modulus values when compared with BSAMA counterparts, and for the 10% BGNP scaffolds, a 3.7‐fold increase was obtained. The controls without BGNP had lower Young's modulus for both matrices. The bare BGNP control for HAMA exhibited the largest modulus (79 ± 52 kPa). Nonetheless, it also presented the largest standard deviation among all tested formulations, which attested to their heterogeneity. This was not observed for the BSAMA bare control. These results reflected that particle inclusion could increase scaffold stiffness, as per the case for HAMA, but also decrease, as was seen for BSAMA scaffolds. Similar findings have been previously reported for composites with similar natures, where particle inclusion can both be an adjuvant for increasing mechanical robustness, but can also destabilize matrix networks.^[^ ^19^, ^49^, ^50^ ^]^ Moreover, these findings can attest to the organic/inorganic phase interaction ability to stabilize and increase the scaffold's mechanical properties.^[^ ^19^, ^49^, ^50^ ^]^

Figure 4C presents the toughness of the same scaffolds, that is, the amount of energy they could withstand before rupture. HAMA scaffolds showcased the highest values between the two matrices. It was observed that toughness decreases with increasing amounts of BGNP, for both matrices. There was a small relation between acquired stiffness and the scaffolds’ brittleness with the inclusion of higher particle amounts. The 0% BGNP control scaffolds had the highest toughness (25 ± 16 kJ m^−3^ for HAMA and 7 ± 2 kJ m^−3^ for BSAMA scaffolds). For the 5% and 10% BGNP scaffolds, respectively, HAMA had the highest values with 22 ± 5 and 16 ± 3 kJ m^−3^. BSAMA scaffolds had 6 ± 1 and 4 ± 1 kJ m^−3^, for the same BGNP %, respectively. This decrease in toughness was expected to result from the covalent crosslinking between the NC phases. Yet, without the primary chemical crosslinking, in the bare BGNP controls, for HAMA, toughness reached ≈11 ± 3 kJ m^−3^, indicating that the absence of interfacial interaction can also hinder the toughness of the scaffold. Bare BGNP heterogeneous dispersion (as previously seen in Figure 2E) and low bond to the matrix did not redeem reinforcing properties. With the primary chemical crosslinking, the toughness of the HAMA scaffolds increased almost 1.5‐fold in comparison to the control. In BSAMA scaffolds, this effect was not exhibited, which we hypothesize being due to the larger impact the secondary matrix crosslinking had on the scaffold's mechanical properties, as previously demonstrated.

Regarding the maximum compressive stress the scaffolds could withstand before collapsing (Figure 4D), the tendency was similar to that of toughness and likely related. BGNP addition resulted in lower maximum endured compressive forces. HAMA scaffolds outperformed, where for the 5% BGNP scaffolds, HAMA reached almost four times more compressive stress than BSAMA (192 ± 57 kPa compared to 50 ± 10 kPa). The ultimate stresses were lower for the 10% BGNP scaffolds, with 159 ± 36 kPa for HAMA, which was still five times higher than the BSAMA scaffolds could withstand (30 ± 9 kPa). 0% BGNP controls could withstand 230 ± 147 kPa for HAMA and 48 ± 8 kPa for BSAMA scaffolds. The bare BGNP controls had the lowest values of 119 ± 33 kPa for HAMA and 39 ± 41 kPa for BSAMA. These values suggest that the phase interaction could help the scaffolds withstand more compressive stress. This was especially noticeable in the HAMA formulations, which could only rely on this interaction.

Ultimate strain was also evaluated, as the maximum strain the scaffolds could endure until breakage (Figure S5A, Supporting Information). Here, the matrices inverted their behavior, where BSAMA was able to sustain higher strains and was considered the most ductile. BSAMA NC with 5 and 10% BGNP had similar ultimate strain values of 58 ± 7 and 56 ± 13%, respectively. HAMA counterparts presented values of 44 ± 3 and 41 ± 4%, respectively. For HAMA, the 0% BGNP formulation had ultimate strains of 43 ± 4%, similar to that of the NC scaffolds, whereas for the control without the primary crosslinking, the maximum reached strain was only 30 ± 6%. For the case of BSAMA controls, the 0% BGNP formulation reached the highest ultimate strain of 68 ± 17%, whereas the bare BGNP formulation reached a lower value of 47 ± 25%. Yet, we could conclude that the interfacial interaction helps the scaffolds dissipate mechanical energy more effectively under load, as exhibited when compared with the ultimate strain of bare and 10% BGNP formulations, which had the same percentage of added fillers.

BGNP addition could generate stiffer NC scaffolds, where a trade‐off was observed between acquired stiffness and ductility of the scaffolds. The primary crosslinking‐enabled phase interaction showcased a similar effect, more noticeable in the HAMA formulations. BGNP inclusion without this crosslinking resulted in heterogeneous scaffolds with higher outcome deviations, which underpinned its importance. For the BSAMA formulations, which were sensitive to the EDC/NHS‐mediated crosslinking on the matrix, the NC phases’ crosslinking did not exhibit a significant effect, apart from the heterogeneity and huge deviation in values. For both matrices, BGNP inclusion and matrix crosslinking did not impart very significant differences throughout the mechanical testing of the scaffolds. This was an outcome that had been previously reported in other works.^[^ ^49^, ^51^ ^]^ It was evidenced that both matrices could benefit from the ink engineering strategy to produce homogeneous scaffolds with the same inorganic fillers. The primary crosslinking could effectively equip better particle dispersion and mechanical properties when compared with the same formulations without primary crosslinking. Comparing mechanical performances, HAMA NC could better serve as a load‐bearing, stiffer scaffold, desirable for applications needing structural support. BSAMA scaffolds’ employment could be more consistent with applications where ductility is also paramount. Indeed, these matrices have already been used in osteogenic and chondrogenic TE, including in composite formulations.^[^ ^28^, ^52^, ^53^, ^54^ ^]^

#### 2.3.2 Gradient Nanocomposite Constructs

The mechanical properties of the combination of these formulations in gradient constructs were also assessed. Both HAMA and BSAMA constructs with 0, 5, and 10% BGNP layers were produced and mechanically tested in the same conditions as the individual formulations. For these constructs, each layer had the same size and volume as its individual scaffold counterpart. The results of the mechanical testing are presented in Figure 4E–G, and Figure S5C (Supporting Information), for Young's modulus, toughness, ultimate stress, and ultimate strain of the gradient constructs, respectively. Figures 4H and Figure S5B (Supporting Information) present gradient constructs, and the cross‐sectional view of a HAMA gradient constructs, respectively. These results showed a cohesive and stable nature with defined but integrated layers. Figure S5D (Supporting Information) showcases the loading and compression of a BSAMA gradient constructs, and Figure 4I displays the stress/strain graphs of all the replicas used for compressive testing.

The results showed that HAMA gradient constructs present higher Young's modulus, toughness, and ultimate stress values than BSAMA gradient constructs. The ultimate strains, although not statistically significant, were higher for BSAMA, as also previously seen in individual scaffolds. The layered addition of the different formulations in a single scaffold resulted in a Young's modulus 2.5 times higher for the HAMA gradient constructs (28 ± 6 and 11 ± 3 kPa, for HAMA and BSAMA constructs, respectively), which was also in line with the previous results. The toughness of the HAMA constructs was also higher, being able to withstand 9 ± 3 kJ m^−3^, whereas BSAMA constructs 3 ± 1 kJ m^−3^. The ultimate stress endured for HAMA constructs was ≈65 ± 25 kPa, until breakage, and for BSAMA was ≈23 ± 6 kPa. Between the two gradient constructs, the ultimate strains were similar, of ≈49 ± 5 kPa for BSAMA and 45 ± 5 kPa for HAMA gradient constructs. These results corroborated that HAMA gradient constructs are stiffer and can withstand more compressive stress before breaking, with similar ductility to the BSAMA constructs. The mechanical testing results showed similar properties to what was previously reported by other authors.^[^ ^19^, ^55^, ^56^ ^]^

Gradient constructs have been utilized for their ability to enhance angiogenic and osteogenic induction capabilities.^[^ ^57^ ^]^ Triphasic and multilayered constructs, which typically entail abrupt and substantial changes in mechanical properties and nature between layers, lead to associated layer delamination and subsequent tissue separation, which gradient contructs can mitigate.^[^ ^58^ ^]^ The ink engineering approach explored in this work was able to generate different ink formulations with compatible mechanical properties and the inherent ability to seamlessly integrate different layers through their photocuring. This approach has also demonstrated good results in other reports.^[^ ^57^ ^]^ The integration of BSAMA and HAMA in gradient constructs can mitigate the aforementioned pitfalls of these sought‐after strategies. Further, being able to fine‐tune the BGNP content and nature of the matrix, it can also impart more biological significance to the constructs. Gradient constructs, namely with 3D printed anisotropic gradient‐structured constructs, have already been able to induce zonal‐dependent osteogenic and chondrogenic differentiation and ECM deposition, showcasing the relevance of studying phasic constructs and their interfaces.

#### 2.3.3 Multilayered Multi‐Material Nanocomposite Constructs

##### Mechanical Testing

**Figure 5:**
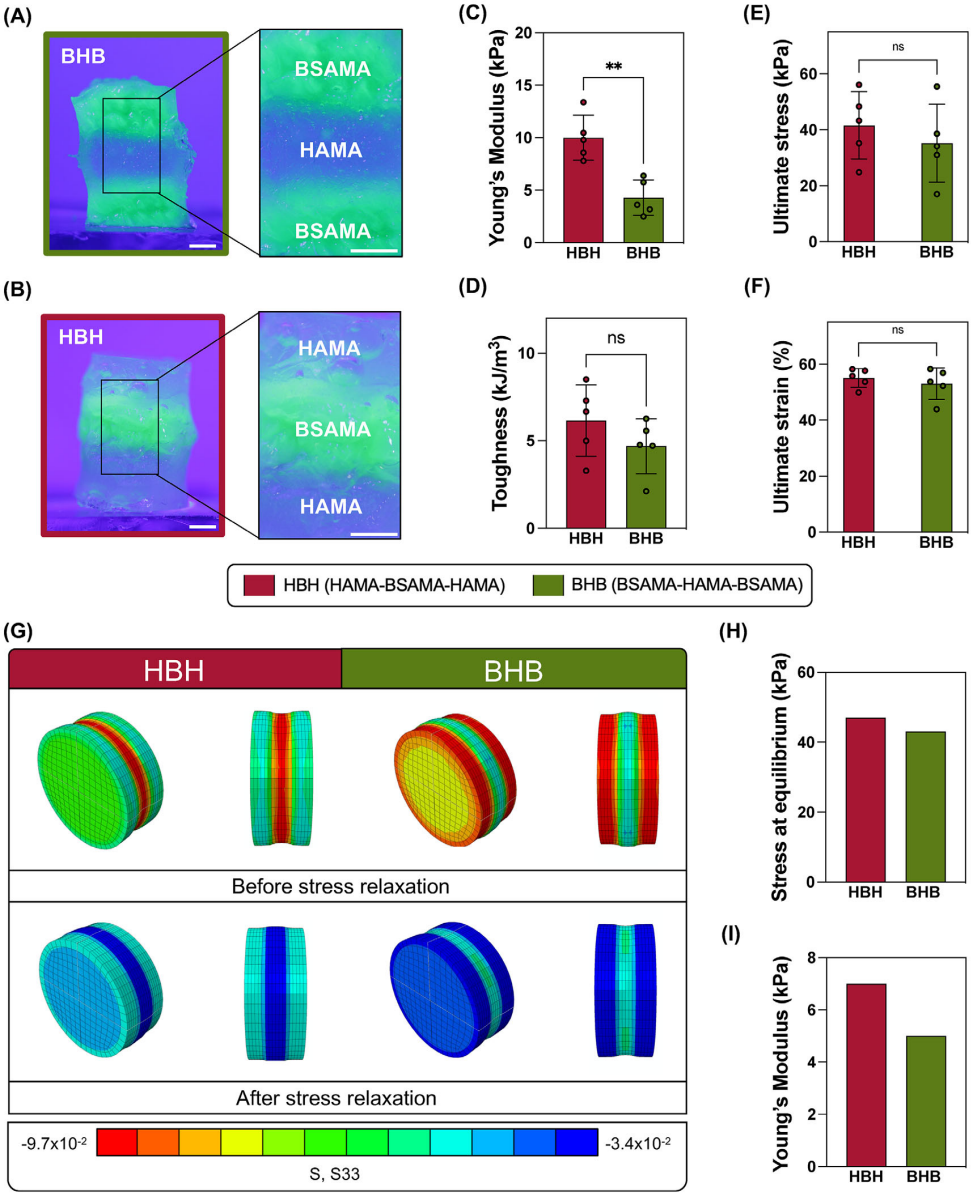
Multi‐material construct mechanical characterization and computational simulation: A) Multi‐material multilayered constructs composed of BSAMA 5% BGNP NC bottom and top layers with HAMA 5% BGNP middle layer. B) Multi‐material multilayered constructs composed of HAMA 5% BGNP NC bottom and top layers with BSAMA 5% BGNP middle layer. In both (A) and (B), the BSAMA NC is stained. C) Young's modulus, D) Toughness, E) Ultimate stress, and F) Ultimate strain values for multi‐material constructs. G) Computational simulation of multi‐material constructs before and after compression. H) Computational simulation of stress relaxation behavior of the multi‐material constructs. I) Computational simulation of the mechanical properties of multi‐material constructs. Scale bars: 2 mm.

Given the previously discussed mechanical characterization results and accounting for the individual potential of both matrices, we studied the employment of BSAMA and HAMA NC in multi‐material constructs. 5% BGNP NC inks were selected for these assays due to their adequate rheological and mechanical performance in both matrices. **Figure** **5**A,B showcases the two tested constructs, which are composed of three alternating layers, where the middle layer is of either BSAMA (represented as HBH) or HAMA (represented as BHB). The volume fraction of each layer is 33% of the total construct. We produced these constructs taking into consideration that each layer should have the same size and volume as its individual scaffold counterpart. From these images and the handling of the constructs, we could perceive that the layers were, in fact, seamlessly integrated. This was related to the methacrylic moieties that both matrices possess, which were leveraged to photocure and covalently crosslink layers of different matrices. Figure 5C–F represent the Young's modulus, toughness, ultimate stress, and ultimate strain of both multilayered constructs, respectively.

Regarding the Young's modulus of these constructs, HBH showcased the highest values (10 ± 2 kPa) compared to BHB (4 ± 2 kPa). This was the only statistically significant difference between the analyzed properties of the multi‐material constructs. The toughness of the constructs was slightly higher for HBH, which could withstand 6 ± 2 kJ m^−3^, while BHB could withstand ≈5 ± 2 kJ m^−3^. The HBH constructs broke at ≈42 ± 12 and 55 ± 3% of strain, whereas BHB at 35 ± 14 kPa and 53 ± 6%. The HBH constructs showcased a stiffer behavior with toughness and ultimate stress and strains similar to that of BHB without statistical significance. Although the HBH constructs had a larger volume fraction of HAMA NC (which has higher mechanical performance), the inclusion of BSAMA NC could result in constructs of similar ductility as those of BHB with half the volume fraction. Additionally, compared with their individual counterparts or even to the gradient constructs, there was a decrease in values obtained for Young's modulus and toughness, especially on BSAMA‐based constructs. Other works depicting multilayered constructs composed of layers of different mechanical properties have demonstrated similar behavior. The multi‐material constructs’ mechanical properties are diminished in comparison to their individual components’ properties.^[^ ^59^ ^]^ The similar ultimate stress and strain values for both multi‐material constructs, and also their similarity or even increase regarding the BSAMA NC individual counterparts or gradient constructs, are indicative of the seamless and successful layer integration, as well as of the beneficial effect of the addition of HAMA for protein matrix reinforcement.

The possibility of creating multilayered and multi‐material constructs with seamless layer integration is paramount in multi‐tissue regeneration. With the established tunability of the inks regarding rheological, mechanical, and bioactivity properties, applicability can be broadened.

##### Computational Simulation

Given the satisfactory results for the integration and mechanical properties of both inks in multi‐material constructs, a finite element simulation was explored to sustain the applicability of these constructs for multi‐tissue bioengineering. An isotropic visco‐hyperelastic model was employed to numerically characterize the mechanical response of BSAMA and HAMA. The visco‐hyperelastic parameters were obtained and optimized through a micro‐genetic algorithm. This calibration was performed in two steps: i) calibration of the viscous properties using the normalized relaxation curve from the experimental relaxation phase, making the process independent of unknown hyperelastic properties (Figure S6E,F, Supporting Information); ii) calibration of the hyperelastic parameters, using the absolute values of the experimental curve, since the viscous parameters are already determined (Figure S6G,H, Supporting Information). Errors smaller than 5% were calculated for both constructs, indicating a good fit of the experimental data and numerical curve. The parameters for each hydrogel resulting from the optimization process are reported in Table S1 (Supporting Information).

Figure S6A,B (Supporting Information) present the stress‐relaxation curves at (lower) 33% strain, and Figure S6C,D (Supporting Information) present the stress‐relaxation curves at (higher) 50% strain, for both BSAMA and HAMA 5% BGNP NC, respectively. Stress‐relaxing properties mean that, under constant strain, the constructs can gradually decrease in stress. This property is paramount and often associated with load‐bearing capability in dynamic environments. It is also important for TE, where it represents better tissue mimicry and can influence cellular responses, as human tissues are also highly viscoelastic.^[^ ^60^, ^61^ ^]^ Viscoelastic hydrogels possess an elastic component that immediately responds to the compressive stress, while the viscous part slowly rearranges, reducing stress over time. Our results showcased that BSAMA had a much higher stress‐relaxing capacity than HAMA NC scaffolds. Between the lower and higher strains applied, the difference lied in the maximum compressive stress that scaffolds experience. This translated to quicker recovery times for BSAMA. HAMA scaffolds showcased a very small decrease and maintained this same stress throughout, resulting in poor stress‐relaxing properties. Given these results, the necessity of including both NC for a multi‐tissue application becomes more apparent: HAMA could better equip multi‐material constructs with more robust mechanical properties, while BSAMA could give them the necessary stress‐relaxation capacity.

Simplified finite element models of the HBH and BHB multi‐material constructs were developed to simulate mechanical compression until a hold point on multi‐material constructs, providing a complementary validation to our experimental data on single‐component scaffolds. These models had the same construct and layer dimensions as their experimental counterparts, ensuring optimal comparability. Figure S5G (Supporting Information) shows that the BSAMA NC encompassed in multi‐material constructs undergoes significant stress relaxation, contrary to HAMA, which did not exhibit this behavior, corroborating our previous data. Depending on the clinical application, this observation may guide scaffold material selection and placement, where stress relaxation properties are important to avoid excessive compression and stimulate soft tissue regeneration with smaller mechanical stress. Alternatively, excessive stress relaxation can reduce the constructs’ load‐bearing capacity by diminishing its structural stiffness.^[^ ^62^ ^]^ Multilayer constructs can be particularly beneficial in clinical scenarios demanding a combination of efficient viscoelastic behaviors and high mechanical strength. The best option between HBH and BHB will depend on the specific clinical application: BHB could be preferable for applications requiring strong mechanical support at the center combined with effective interfacing with soft tissue. It could be useful, for instance, in large soft tissue defects that need a strong scaffolding system in the center to avoid collapsing. Conversely, HBH could be a better option when the scaffold interfaces with hard tissues, such as in bone tissue regeneration, where HA – being the major component of bone tissue ECM – and BGNP can guide effective bone binding and remodeling, while at the same time BSA can improve multi‐cellular differentiation and functional tissue regeneration in the center, due to its stress relaxing properties.

To provide experimental validation to the numerical simulations, the Young's modulus, measured at the initial region of the compression curve, and the stress at equilibrium values at the end of the relaxation step were calculated (Figure 5H,I) from the simulated stress relaxation curves of BHB and HBH (Figure S6I, Supporting Information). Similarly to the experimental results (Figure 5C), HBH exhibited a slightly larger Young's Modulus than BHB at the initial linear region of the experimental compression curve. Nonetheless, it should be noted that the constitutive model does not account for fracture behavior; therefore, the comparison between the experimental ultimate stress and the numerical maximum stress should be interpreted with caution.

### 2.4 3D Printing of the Nanocomposite Inks

#### 2.4.1 Printability Evaluation

**Figure 6:**
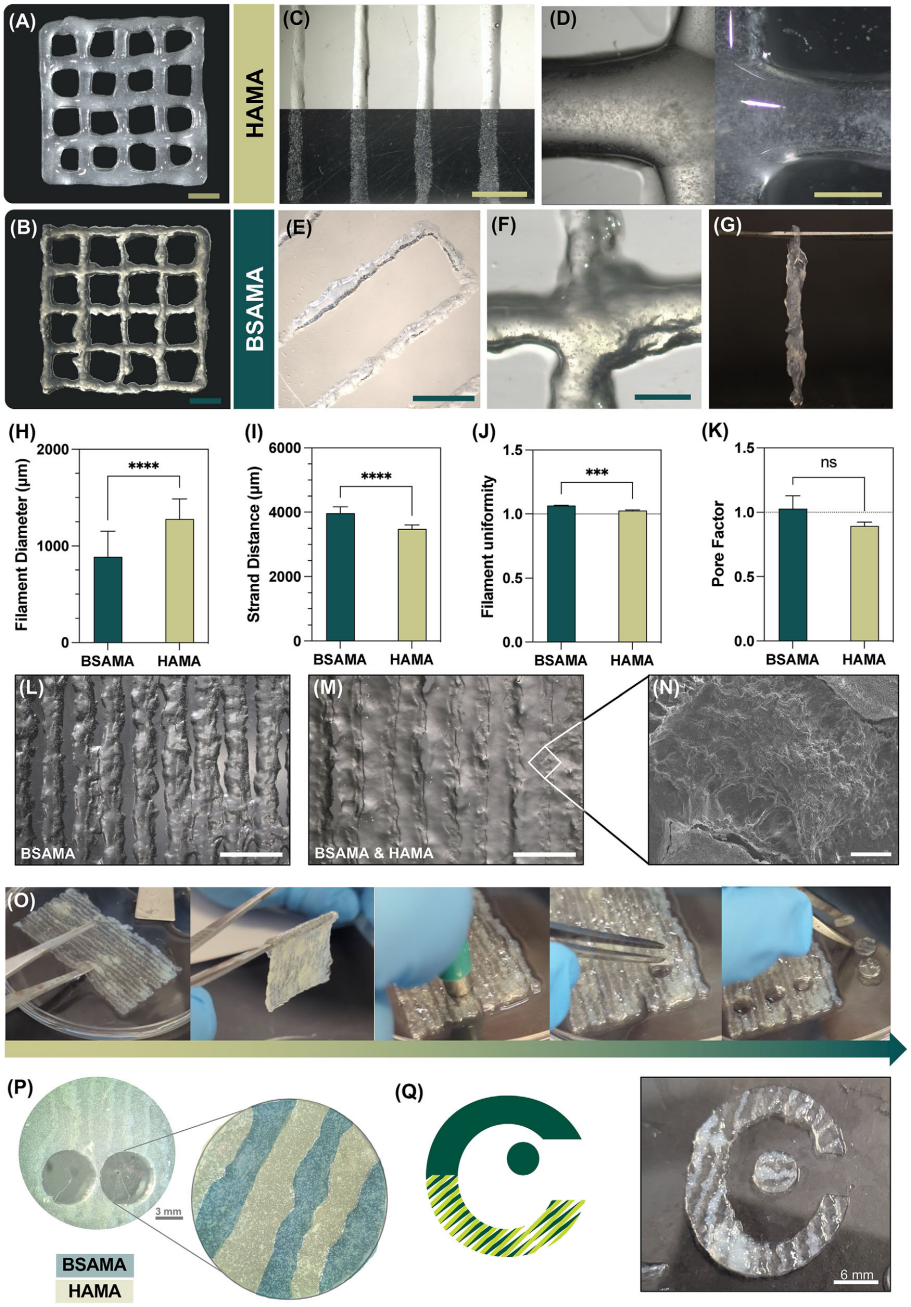
Printability of 5% BGNP HAMA and BSAMA NC inks and multi‐material constructs: A,B) 4‐layer HAMA and BSAMA 20 × 20 mm grids, respectively. scale bars: 4 mm. C) HAMA filaments. scale bar: 4 mm. D) HAMA grid cross‐section. Scale bar: 1 mm. E) BSAMA filaments. Scale bar: 4 mm. F) BSAMA grid cross‐section. Scale bar: 2 mm. G) BSAMA grid handling. H) Filament diameter, I) Strand distance, J) Filament uniformity, and K) Pore factor, which were measured from the grid's composing filaments presented in (A) and (B). L,M) Microscopy images of the BSAMA and HAMA multi‐material construct. Scale bars: 400 µm. N) SEM microscopy image of the interface between layers. Scale bar: 400 µm. O) Workflow to produce the constructs for cytocompatibility assays from surrogate multi‐material construct handling, templating of the 6 mm constructs, and their retrieval. P) 6 mm in diameter construct for the cytocompatibility assays, retrieved from the surrogate constructs. Q) Templated constructs from the surrogate construct, showcasing the COMPASS research group logo.

Multi‐tissue defects are usually irregular in size and architecture, demanding advanced biofabrication techniques for recreating personalized substitutes. Given that the rheological and mechanical properties of HAMA and BSAMA NC inks were suitable for 3D printing applications, printability evaluation was performed for 5% BGNP NC for both BSAMA and HAMA. The print parameters were optimized according to multifarious analysis of the inks’ performance, and considering the displayed pressure dependency, which is typically found in natural‐based inks.^[^ ^38^, ^63^ ^]^ **Figure** **6**A,B present HAMA and BSAMA printed grids, respectively, both composed of 4 layers, which showcased good print fidelity without filament breakage throughout. Figure 6C,E exhibit individual filaments utilized for printing fidelity assessment, while Figure 6D,F display a zoomed‐in crossing point of these grids, both for HAMA and BSAMA inks, respectively, which do not display filament dragging. Figure 6G denotes the handling capability of one BSAMA grid, which showcases the robustness of the printed constructs after secondary post‐printing crosslinking.

The filament diameter, which depicts the size of the extruded filaments, is portrayed in Figure 6H. The BSAMA ink demonstrated the best filament fidelity results, with a mean diameter of ≈889 ± 263 µm, while HAMA inks were ≈1279 ± 208 µm. Nevertheless, both had a significantly higher final diameter compared to the gauge used (410 µm). In turn, the strand distance between filaments, which, concerning the used G‐code, should be 4000 µm, showcased appropriate results; both compared to the theoretical and considering the filament diameter. For the BSAMA filaments, the strand distance was ≈3973 ± 196 µm, while for HAMA inks it was ≈3484 ± 124 µm (Figure 6I). Because BSAMA had thinner filaments, the strand distance was higher, and the opposite happened for HAMA NC inks. Both filament diameter and strand distance exhibited statistical differences between BSAMA and HAMA inks, which again confirmed their different printability profiles. Filament uniformity, represented in Figure 6 (J), which assesses the consistency of the filament's shape and quality, could also indicate the different printability of the two inks. Here, the closest value to 1 reveals an ideal filament uniformity. Both inks showcased good filament uniformity. BSAMA had the furthest value, ≈1.07, revealing a less uniform filament deposition than that of HAMA NC inks, which was ≈1.02, also corroborating the macroscopic evaluation. This could indicate that although the HAMA filaments had higher diameters, their deposition was more uniform. On the contrary, BSAMA inks generated thinner extruded filaments with less uniformity. The pore factor – or printability index – was evaluated on the printed grids (Figure 6K). Pore factor values over 1 often predict over‐crosslinked inks, which generate irregular pores, whereas values under 1 are typically under‐crosslinked inks, which convey rounder pores. Values closest to 1 predict inks with appropriate properties and optimized print parameters, which can print structures closest to the employed G‐codes.^[^ ^63^ ^]^ Both grids showcased appropriate pore factor values close to the optimal, with no statistical difference between the two. However, the difference in values (1.03 ± 0.1 for BSAMA and 0.89 ± 0.03 for HAMA) corroborated once more the different printability profiles of the two inks, resulting in scaffolds with filaments that are thinner but also more irregular and overly crosslinked for BSAMA.

The rheological characterization of the inks could also predict and corroborate the printability outcomes. Both shear‐thinning ink formulations exhibited low viscosity at mid‐to‐high shears (Figure 3A,B) and high yield points (Figure S3B,F, Supporting Information), consistent with good extrudability and filament deposition outcomes (Figure 3; Figure S4, Supporting Information). Regarding the printability evaluation, both inks could be extruded under low applied pressure, corroborating the rheological characterization. Further, the wide linear viscoelastic regions of both inks (Figure S3B,F, Supporting Information) were consistent with good structure retention and filament stability. The loss tangent rheological data (Figure 3E), which showcased elastic‐dominant inks (0 < tan(δ) < 1), was consistent with shape retention after extrusion. When comparing the loss tangent values of both inks, their printability outcomes are aligned. BSAMA, which had the lower loss tangent (0.24), showcased less uniform filaments but also the least filament spreading ratio. On the contrary, HAMA inks (0.46) printed thicker (more spread) yet more uniform filaments (Figure 6H,J). Ultimately, these features also influence construct printability, imparting, in this case, different pore factor values, although no statistical differences were found. Both inks lacked shape retention, leading to higher filament diameter values, which is also indicative of their thixotropic behavior, not being able to recover their initial viscosity after being subjected to high stresses.^[^ ^64^ ^]^ While this can imply the need for applying lower extrusion pressures during printing, a compromise must be made between printing fidelity and structural integrity of the printed structures. Also, the prevalent elastic nature of the scaffolds under a range of frequencies (Figure S3A,G, Supporting Information) was also consistent with the robustness of the printed constructs, as well as their ability to sustain proper layer stacking after photocrosslinking. Overall, both inks showcased complementary printability and rheological profiles, which could be leveraged for 3D printing multi‐material constructs (Figure 6O). These were performed by printing in first place the BSAMA inks (low spreading) and by second the HAMA inks, which could effortlessly fill the voids of the construct (high spreading), as previously observed. Ultimately, our printability results corroborated the inks’ rheological characterization, which was leveraged to generate multi‐material constructs with good print fidelity.

Both inks could generate constructs with good print fidelity, printable at considerably low pressures. In combination with the rheological characterization and mechanical testing of the single, gradient, and multi‐material constructs, this could inform on the feasibility of employing these engineered inks for multi‐tissue biomedical applications. Yet, as previously reported, the primary crosslinking strategy was determined by the available reactive groups.^[^ ^19^, ^38^ ^]^ This was also dependent on batch‐to‐batch variability of the matrices and fillers, as well as the reproducibility of the crosslinking, phenomena that can impact printability and print fidelity. Nonetheless, our approach may be improved with a primary crosslinking strategy including dynamic bonds to instigate faster viscosity recovery and self‐healing properties. Regarding the secondary crosslinking strategy, it could encompass faster photocuring methods and/or in situ photocuring to ensure a better print performance.^[^ ^65^, ^66^, ^67^, ^68^, ^69^ ^]^ Yet, when dealing with natural‐based materials, the necessary complex synthesis/modification methodologies can interfere with their innate bioactivity. The aforesaid alternatives can also affect reproducibility, scalability, and variability concerns.

#### 2.4.2 3D Printing of Multi‐Material Constructs

To further attest to the printability and feasibility of employment of these engineered nanocomposite inks, simple multi‐material constructs were explored. These constructs were produced to both evaluate the feasibility of printing both inks simultaneously using two pneumatic printheads and to be used for cytocompatibility evaluation of the multi‐material systems. A square 36 cm^2^ construct was designed with intercalated filaments of BSAMA and HAMA NC inks, which were designed with a 1 mm in diameter. The G‐code used for the printing process is depicted in Figure S7A (Supporting Information). This diameter was chosen according to the printability results, and the printing parameters were optimized to accommodate these structures.

Figure 6L,M depicts micrographs of the printing workflow: first, there is the printing of the BSAMA ink, followed by the HAMA ink, intercalating the filaments of both materials. The printing of the BSAMA ink is also represented in Figure S7B (Supporting Information). Figure 6N represents the SEM microscopy image of the multi‐material construct, in which the interface between the two inks can be seen, where, in this case, the BSAMA ink was in the middle. Figure 6O presents the workflow to produce the multi‐material constructs. Here, after both BSAMA and HAMA NC inks printing, the construct was used as a surrogate, from which 6 mm diameter round constructs were cut and utilized for cytocompatibility assessment (Figure 6P). The robustness and handling of the surrogate constructs were also demonstrated and corroborated the seamless integration of both NC inks. Figure 6Q illustrates the potential for more intricate designs that could be generated from the surrogate construct, namely through the use of templates.

The seamless combination of both inks in multi‐material constructs is of utmost interest for biomedical applications and TE approaches. For extrusion 3D printing, there is a lack of printable BSA‐based inks, where, for biomedical applications, other strategies apply BSA coatings for better cytocompatibility and bioactivity results.^[^ ^70^ ^]^ It is most often used as a supplement or surface modifier for enhancing scaffolds’ cell attachment, adhesion, proliferation, and osteogenic differentiation. Being able to employ these natural‐based materials as a printable formulation is paramount for TE, which so far has led to substandard results. On the other hand, HA‐based biomaterials often do not favor cell attachment and new tissue formation, being often utilized as a viscosity and mechanical property enhancer. Nevertheless, other authors have found that HAMA promotes osteogenic differentiation, where the addition of bioactive osteoconductive fillers can induce bone defect regeneration.^[^ ^71^ ^]^ Further, HAMA NC scaffolds have shown mechanical properties similar to those required for achieving bone tissue regeneration.^[^ ^72^ ^]^ Previous studies have found that soft scaffolds (≈1 kPa) can promote adipogenesis, slightly stiffer matrices (≈10 kPa) can promote myogenesis, and more rigid scaffolds (≈100 kPa) can promote osteogenesis.^[^ ^73^, ^74^, ^75^ ^]^ Thus, the differential characteristics of the developed inks developed manuscript hint at their broad applicability, namely for bone‐to‐cartilage TE.

### 2.5 Cytocompatibility of Multi‐Material Constructs

The multi‐material constructs (Figure 6P) were subjected to cytocompatibility testing by seeding human adipose stem cells (hASCs) on top of the constructs, which were cultured for seven days. During this period, the metabolic activity was evaluated with Alamar Blue for both the multi‐material constructs and bulk individual HAMA and BSAMA NC. The multi‐material constructs were also simultaneously subjected at days 1 and 7 of culture to qualitative analysis of cell proliferation via calcein and propidium iodine (PI) Live/Dead staining, and cell morphology was evaluated with 4′,6‐diamidino‐2‐phenylindole (DAPI) and phalloidin staining. Our main goal was to assess a possible preference toward one of the materials, opening possibilities for creating multi‐material constructs that could instruct cells toward specific paths.

**Figure 7:**
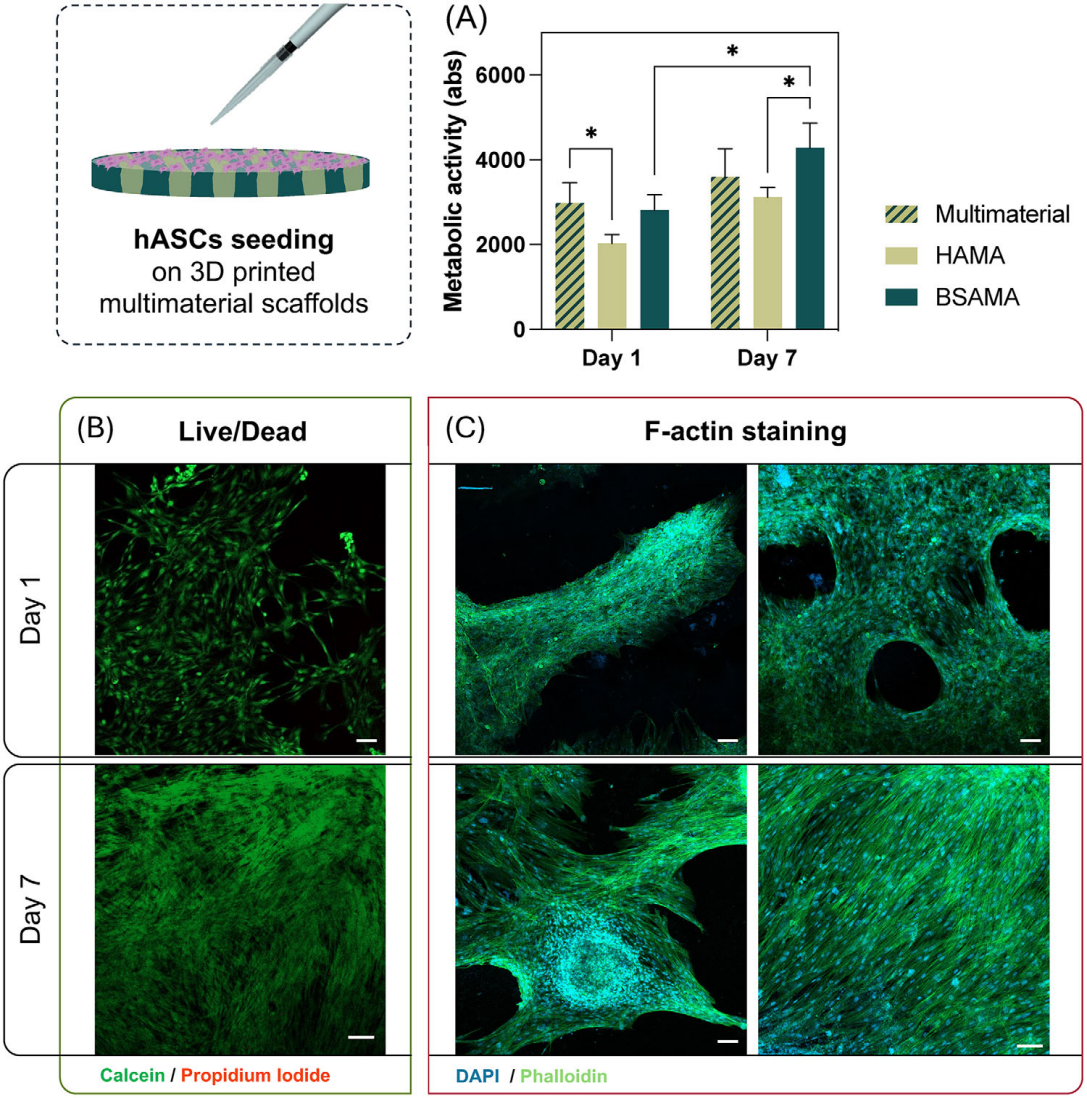
Cytocompatibility assay: A) AlamarBlue metabolic activity assay of cell‐seeded multi‐material and BSAMA and HAMA NC constructs. B) Fluorescence microscopy images of the live/dead staining assay of the multi‐material constructs. C) Fluorescence microscopy images of DAPI/Phalloidin staining assay of the multi‐material constructs. Scale bars: 100 µm.

The metabolic activity assessment data can be observed in **Figure** **7**A, which showcases a continued trend of increasing metabolic activity from days 1 to 7 in all constructs. Only BSAMA NC constructs showcased statistical differences between days 1 and 7. On day 1, the multi‐material constructs had the highest metabolic activity values, being statistically significant when compared to the HAMA NC scaffolds. BSAMA NC scaffolds’ metabolic activity was similar to the multi‐material constructs throughout. On day 7, HAMA NC scaffolds remained with the lowest absolute values, whereas BSAMA NC scaffolds had the highest increase in metabolic activity, followed by the multi‐material constructs. When comparing the cellular metabolic activity of BSAMA NC, HAMA NC, and the multi‐material constructs, there was a clear disparity between the cells seeded on HAMA NC scaffolds and the remaining ones. These results suggest improved cytocompatibility of BSAMA NC, whose inclusion in the multi‐material constructs allowed for similar results, with the added mechanical benefit of HAMA NC ink presence. This was likely attributed to the proteinaceous nature of the ink and presence of cell attachment motifs, which also explains the similar behavior on the multi‐material constructs. In comparison, HAMA underperformance in supporting cell metabolic activity was likely the result of its higher crosslinking density and lack of innate cell adhesion motifs.^[^ ^76^ ^]^

To further clarify these results, we proceeded to qualitative analysis. Figure 7B presents the Live/Dead staining performed on the multi‐material constructs. There was a noticeable surge in the number of green‐stained cells throughout culture time. Cell adherence was also successfully evidenced throughout the constructs, although much more pronounced in the BSAMA NC scaffold‐composing filaments. Cell attachment was confirmed since day 1 with noticeable hASCs proliferation to the whole constructs on day 7. From the Live/Dead staining assay, there were minimal to no dead cells, which is an indicator of the non‐cytotoxicity and suitability of these constructs and both inks for biomedical applications.

DAPI/Phalloidin staining assay (Figure 7C) was performed to visualize cell morphology and placement. Because of the autofluorescence or BSAMA NC inks and the composition of BGNP of both inks, a proper staining visualization was hampered. The results evidenced cell elongation since day 1, with minimal rounded cells. Similar to what was seen in the Live/Dead staining, cells could proliferate throughout the constructs. Especially on day 1, cell adherence was mainly seen on the BSAMA NC sections of the constructs, being guided by the printed filaments. In some sections, as can be seen in Figure 7C, cells could form bridges between the BSAMA filaments, further suggesting that these constructs could guide cell attachment and proliferation. Further, on day 7, the multi‐material NC had high cell counts due to the extensive sprouting of cells to the HAMA NC filament sections of the constructs, in some cases even populating the whole region of the constructs (as seen below). This type of preference for the BSAMA NC has already been reported and exploited for cell recruitment and increased cell attachment capability of serum albumin for TE approaches.^[^ ^77^, ^78^ ^]^ HAMA's cytocompatibility in the printed constructs and the individual constructs has also showcased similar results to those of other works.^[^ ^28^, ^79^ ^]^

Overall, these results sustained the importance of multi‐material constructs, which could benefit from the different nature of the materials used, which imparted several added features. Given that this assay was performed in dynamic conditions, we could conclude that 3D printing allowed for spatial control over cell attachment and, consequently, proliferation, in this case due to the placement of the BSAMA NC filaments of the constructs, which were preferred by the cells for that purpose. Given that BSAMA showcased this important stress‐relaxing capability, in contrast to HAMA, we hypothesize that this feature also had an important weight on our results. These results have demonstrated the cytocompatibility of both inks and multi‐material constructs, not only confirming their feasibility and utility on 3D printing methodologies for biomedical applications but also denoting the importance of developing multi‐material combinations, enabling including cell niches and specific cell guidance patterns that could be extremely important and useful in the creation of a tailored therapy. In the future, assessment of changes in cell phenotype in longer timeframes is proposed, not only in these intercalated multi‐material formulations, but also in more intricate printing patterns. Also, the inclusion of other cell types would be interesting to assess the preference and morphogenesis of different cells toward specific stress‐relaxing profiles of the evaluated materials, as previously demonstrated.^[^ ^80^, ^81^, ^82^, ^83^, ^84^ ^]^

### 2.6 Computer‐Simulated Biomedical Applicability

**Figure 8:**
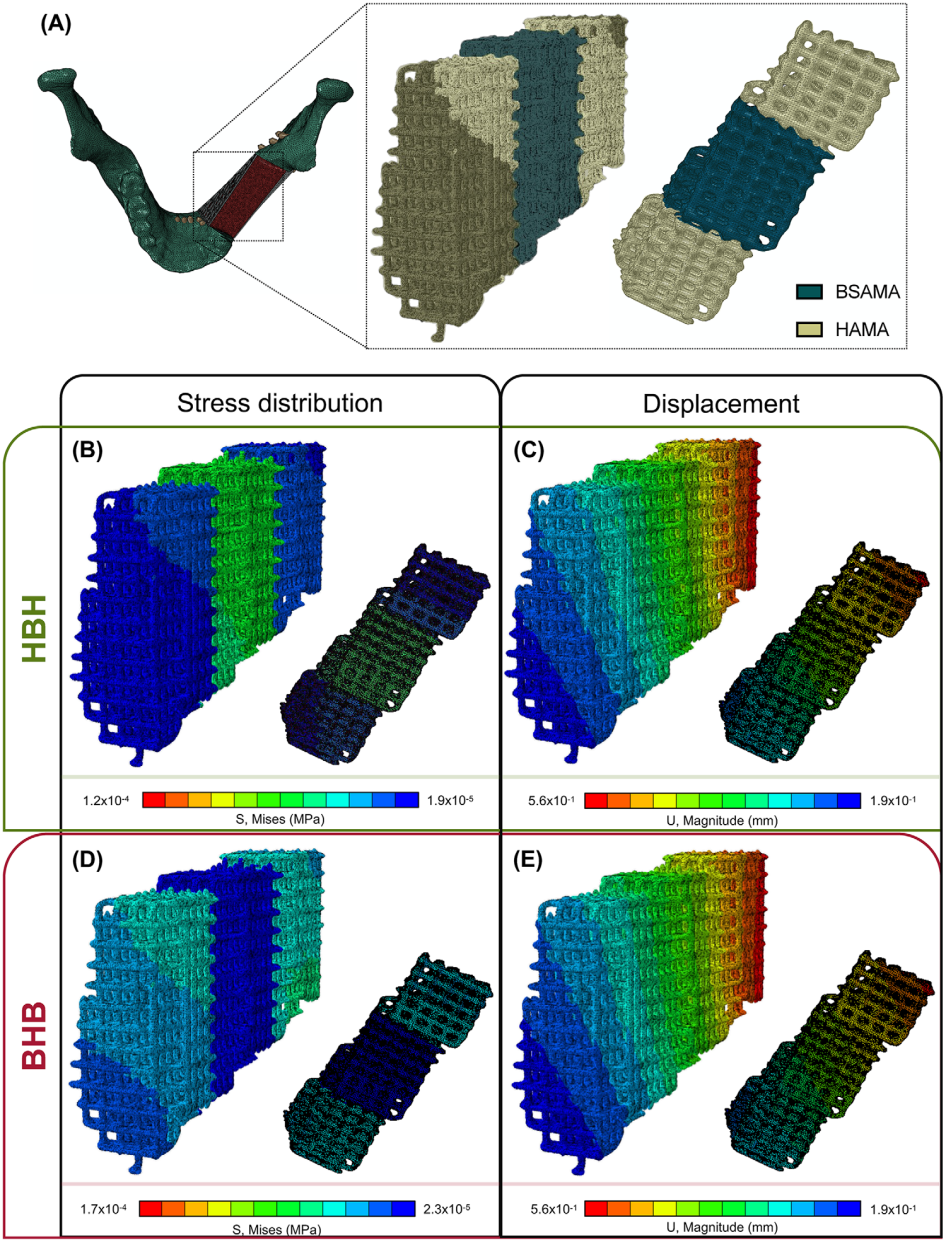
A) Numerical model of the human mandible and different perspective examples of the multi‐material construct to be inserted into the mandibular joint. B,C) Von Mises stress distribution and displacement of HBH at the end of the numerical simulation, respectively. D,E) Von Mises stress distribution and displacement of BHB at the end of the numerical simulation, respectively.

A previously developed numerical model of a mandible was used to evaluate the mechanical performance of the multi‐material constructs, highlighting the important interconnection between experimental, numerical, and clinical fields.^[^ ^85^ ^]^ The mandibular model, illustrated in **Figure** **8**A, had a defect in order to test the multi‐material constructs as illustrated. Both BHB and HBH constructs were implanted into the mandibular joint simulated defect, and the resulting stresses were compared between them. Unilateral clenching was selected as the sole task to be tested, based on findings from several studies indicating that contralateral molar occlusion generates the highest mechanical loading, thereby representing a critical clenching scenario. The multi‐material construct was created using a combination of MATLAB and Blender software. MATLAB was employed to generate a G‐code file, providing creative flexibility for designing custom construct architectures. Additional information regarding the components of the model, materials and parameters, loads, contact, and boundary conditions can be found in previously published literature.^[^ ^85^ ^]^

The von Mises stress distributions and displacements of HBH and BHB are illustrated in Figure 8B–E. In terms of stresses generated during the clenching simulation, the HBH construct presented smaller stresses than BHB, while also exhibiting different stress distributions. This observation indicates that having the BSAMA NC layer between the HAMA NC layers provided greater mechanical stability to the multi‐material construct, which corroborated initial observations on the mechanical characterization of these constructs. It also supported the idea that having HAMA in direct contact with the mandibular bone was more advantageous, as it exhibited a lower viscous response. This could be observed when comparing Figure 8B,D. In terms of displacement, HBH and BHB did not exhibit significant differences, which suggested that both construct configurations would not negatively affect the kinematics of the construct during mandibular clenching (Figure 8C,E), being a highly satisfactory result for the clinical application of these multi‐material constructs in complex procedures such as a human mandibular reconstruction.

These numerical simulation approaches can also be applied to a different type of multi‐material construct, featuring a hydrogel core, such as BSAMA and/or HAMA NC ink, surrounded by a ceramic construct. This configuration could combine the regenerative advantages of the hydrogel with the enhanced mechanical support provided by the ceramic construct, offering a balanced solution for TE applications. Indeed, a major advantage of employing numerical simulations is the ability to simulate different models (other than the mandible) and different applied forces (other than a chewing motion). Virtually, a variety of constructs can be studied for their physiological relevance and suitability or performance upon implantation. We believe that this pre‐assessment would give very important and relevant data for construct optimizations, enabling the saving money and streamlining the regulatory process for the acceptance of these therapeutic solutions into the market.

## 3 Conclusion

This study presents a reproducible ink engineering strategy aiming to overcome key limitations in the 3D extrusion printing of natural‐based NC inks for multi‐tissue TE. The primary crosslinking strategy provided tunable rheological properties, enabling the formation of mechanically reinforced NC, with enhanced bioactive potential and homogeneously distributed fillers. The BGNP acted as reinforcement agents both rheologically and mechanically, while also serving as platforms for the therapeutic delivery of ions that promote regeneration. Their homogeneous distribution also mitigated common extrusion‐related issues such as needle/nozzle clogging, while enabling the fabrication of complex structures without the use of support baths. Combined with the secondary post‐printing photocuring strategy, this approach enabled spatiotemporal control over the crosslinking of the structures, where the methacrylic moieties of both matrices allowed for their seamless integration. The developed inks showcased the expected bioactivity of their components and allowed the fabrication of constructs with appropriate degradation kinetics, mechanical performance, and 3D printing applicability, allowing the creation of various construct architectures, including multi‐material constructs on both HAMA and BSAMA NC inks. Both experimental and computational approaches were employed to design and evaluate gradient and multi‐material constructs under clinically relevant conditions, a feature that remains underexplored in the current literature. Additionally, the printed multi‐material constructs supported hASCs' proliferation, confirming their cytocompatibility.

Overall, this study presents a proof‐of‐concept platform for processing otherwise unprintable natural‐based materials into bioactive NC inks. The approach supports the incorporation of various (nano) particles into composite formulations for a wide range of tissues and therapeutic targets. Further, the thorough experimental characterization and finite element modelling highlighted its potential to effectively accelerate the development of customized, optimized solutions in regenerative medicine, paving the way for future innovative and personalized therapies aimed at multi‐tissue regeneration.

## 4 Experimental Section

### Bioactive Glass Nanoparticles

The BGNP were synthesized through a modified sol–gel process with co‐precipitation optimized from previous works.^[^ ^19^ ^]^ Three precursors were used: calcium, comprised of 7693 g of calcium nitrate tetrahydrate in 120 mL of distilled water; silica, consisting of 9,83 mL of tetraethyl orthosilicate in 60 mL of absolute ethanol; and phosphate, as 1.078 g of ammonium phosphate dibasic in 1500 mL of distilled water. Briefly, calcium and silica precursors were hydrolyzed for 3 h under agitation, at pH 2 (adjusted with citric acid monohydrate (10%w/v)). The hydrolyzed precursors were then sprayed onto the phosphate precursor at pH 11,5 (adjusted with ammonium hydroxide) and left under 400 rpm for 48 h. After gelation occurred, the gel phase was separated and washed 3 times in Milli Q water and a last time in ethanol, at 20 000 rpm for 20 min. NP were resuspended via sonication every washing cycle and then dried overnight in a 50 °C oven. PEG (20 000 mw) was added at 2%w/v in a 200 mL aqueous solution that underwent ultrasonication for 30 min. After freeze‐drying for 7 to 10 days, the NP were calcinated at 700 °C for 5 h with heating/cooling ramps of 1 °C s^−1^. Finally, the BGNP were grounded, sifted, and stored until functionalization.

Functionalization was performed in reflux at 130 °C for 24 h under a nitrogen atmosphere. BGNP were dispersed in extra dry toluene at a ratio of 4.5 mL per 0.1 g of particles, using ultrasonication in an ice bath with 30‐second on/off pulse cycles for a total duration of 30 min. APTES was then added dropwise, maintaining an excess of 10 µL per 0.1 g of particles. The modified nanoparticles were retrieved by centrifugation, washed four times with ethanol (20 000 rpm, 15 min.), and dried overnight at 50 °C. The pH was adjusted in Milli Q water to 7,4, and after freeze‐drying for 7 to 10 days, the BGNP were sifted and stored.

### Methacrylated Bovine Serum Albumin

BSAMA was synthesized as previously reported.^[^ ^19^, ^39^ ^]^ Briefly, BSA (5% (w/v)) was dissolved at 37 °C and 500 rpm for one hour in a 0.2 m carbonate‐bicarbonate buffer at pH 9. Methacrylic anhydride (10 µL mL^−1^) was added dropwise and left reacting at 37 °C, 500 rpm, for another hour, at the end of which the pH was adjusted to 7.4. This solution was then dialyzed against distilled water, with a membrane cut‐off of 3.5 kDa (snakeskin dialysis tubing, Thermo Fisher Scientific), and water was changed twice daily for five consecutive days. The solutions were then frozen and freeze‐dried for 7 days under sterile conditions and stored.

### Methacrylated Hyaluronic Acid

HAMA was synthesized according to previous studies.^[^ ^40^ ^]^ Briefly, hyaluronic acid (80 000–100 000 Da, 10 mg mL^−1^) was dissolved in 500 mL of deionized water at 500 rpm and RT, overnight. Afterward, the solution was cooled to 4 °C and stirred while gradually adding 1.25% (v/v) of methacrylic anhydride. PH was maintained between 8 and 9, and the reaction was allowed to occur overnight at 4 °C under stirring. The resultant HAMA was precipitated by ethanol addition (3L) and subsequently subjected to centrifugation for 10 min. at 5000 g, followed by desiccation at 35 °C overnight. The dried HAMA was then dissolved in deionized water and then dialyzed (3.5 kDa cut‐off (snakeskin dialysis tubing, Thermo Fisher Scientific)) for 3–4 days at 4 °C, with frequent water exchanges. The resulting solution was then frozen, lyophilized for 7 days under sterile conditions, and stored.

### Nanocomposite Formulation and Post‐Print Photocuring

BSAMA and HAMA were dissolved (10 and 5%w/v, respectively) in PBS with LAP photoinitiator and filtered. The BGNP, suspended in PBS, was sonicated for 15 min. The BGNP were previously sterilized under UV light for two 30‐minute cycles. The EDC and NHS were solubilized in PBS in 100 and 25%w/v concentrations, respectively, and filtered with a sterile syringe filter with a pore size of 0.22 µm. Afterward, aliquots of the BSAMA/HAMA with LAP mixtures were pipetted to Eppendorf tubes to which appropriate amounts of BGNP, EDC, and NHS were added and then placed in a thermomixer at 1500 rpm, 37 °C, for 3 h, protected from light sources. HAMA‐based formulations were prepared with ≈2% w/v of EDC/NHS and BSAMA‐based with 4% w/v. After this reaction period, the primary reticulation has taken place, and the NC was ready to be further photocured. Photoreticulation was processed with a visible light lamp (405 nm, VALOTM GRAND) for two 3200 mW cm^−2^ cycles. For the case of the rheological assessments, a UV lamp (OmniCure S series, Excelitas. USA) was used at the same intensity. In the printing process the BIOXTM bioprinter (CELLINK Bioprinting AB, Sweden). The 405 nm photocrosslinking head was used for 20 s after each printed layer and had an intensity of 2.5 mW cm^−2^.

### Degree of Modification

H^1^‐NMR was performed to assess the DOM BSAMA and HAMA. Herein, BSAMA and HAMA were dissolved at 20 mg mL^−1^ in dimethyl sulfoxide (Fisher Scientific, USA) and in deuterated water (D20), respectively; and 500 µL were pipetted to NMR tubes. The scans were performed at 300.13 MHz using an 18 s relaxation delay and 512 scans (AMX‐300, Bruker, Germany).

For BSAMA the DOM was calculated by dividing the sum of integrals of the methacrylate groups (*δ* ≈ 5.27–5.63 ppm) by the area under the peak relating to aromatic groups of the BSA backbone (*δ* ≈ 7.22 ppm). For HAMA, the DOM was calculated by dividing the integral of the methacrylate proton peak (*δ *≈ 1.8 ppm) by the peak of the HA backbone (*δ* ≈ 1.9 ppm). The acquired spectra were analyzed with MestreNova software.^[^ ^86^ ^]^

### BGNP Size Distribution

BGNP size distribution was analyzed via TEM microscopy and analyzed using FIJI (120 measurements).^[^ ^43^ ^]^ Briefly, samples were prepared by dispersing an ethanolic nanoparticle suspension onto a Formvar carbon‐coated copper grid 200 mesh (Ted Pella, Redding, CA) and analyzed in a Hitachi transmission electron microscope (H‐8100 Hitachi High – Technologies, Tokyo, Japan). Additionally, dynamic light scattering was utilized (zetasizer nano ZS, Malvern Instruments, UK) by dispersing the BGNP in Mili Q water (1 mg mL^−1^) and sonicated for 15 min. and read in a 1 mL plastic cuvette.

### Scaffold Staining

Osteoimage‐stained scaffolds were observed under a high‐resolution laser scanning confocal microscope (LSM 900, Carl Zeiss, Germany).

### Rheological Characterization

Rheological properties were studied operating a Kinexus Lab+ rheometer (Malvern Panalytical, UK) with an 8 mm plane geometry and a 0.5 mm gap. The linear viscoelastic region and yield strain were determined via a strain sweep assay from 0.1 to 100% at 1 Hz. Photorheology time‐sweep was performed at 0.5% strain and 1 Hz with light exposure at 3.2 W cm^−2^ (OmniCure S series, Excelitas. USA) for a total of 5 min. with the beginning of light exposure after 2 min. Shear‐thinning was evaluated using a shear rate ramp ranging from 0.01 to 500 s^−1^. Frequency sweep assays were performed from 0.01 to 100 Hz at a 0.5% strain. Other than for the photorheological assays, all formulations were not exposed to light.

### Ink Extrudability Testing

Ink flow and extrudability testing were performed by extruding the ink formulations through various gauge needles and nozzles (22G, 25G, and 27G). Syringes containing the formulations were placed in a microfluidic pump (Harvard apparatus, USA), and a 150 µL min^−1^ flow rate for 0% and 5% BGNP formulations was used, and 250 µL min^−1^ for the 10% BGNP inks.

### 3D Extrusion Printing

The NC inks were added in 3 mL cartridges and printed in a BIOX bioprinter (CELLINK Bioprinting AB, Sweden) using a 22G nozzle using a pneumatic toolhead. Printing pressures and speeds employed ranged from 40 to 60 kPa and 8 to 12 mm s^−1^, respectively. The scaffolds/constructs were photocured after each layer at 405 nm with an intensity of 25 mW cm^−2^ for 20 s via a photocuring toolhead (CELLINK Bioprinting AB, Sweden).

### Printability Assessment

Printability was evaluated from the printed filaments and grids, from which microscopy images, filament diameter, strand distance, filament uniformity ratio, and pore factor were measured and/or calculated. Filament diameter was calculated as the mean diameter of 20 measurements across three different filaments. Strand distance was considered as the mean of 20 measurements of the distance between three different filaments. Filament uniformity ratio was calculated as the division of the filament perimeter by its length and considered as the mean of 20 calculations. Pore factor was calculated by dividing the second power of the pore perimeter by 16 times the pore area.

### Simulated Body Fluid Assay

In vitro bioactivity assessment was conducted using Kokubo's SBF to verify the formation of an HAp layer on the HAMA and BGNP NC following immersion.^[^ ^87^ ^]^ Briefly, SBF was prepared alongside NC photocured scaffolds (*N* = 3) for each timepoint. Day 0 is the control group not immersed in SBF. Day 7 samples were kept in an incubator at 36.5 °C for 7 days. At this point, the scaffolds were taken out from the SBF, gently washed with ultra‐pure Milli Q water, and dried in the fridge at 4 °C. For surface characterization, the hydrogels were fixed with conductive adhesive tape to a sample holder stub and sputter‐coated with carbon (K950X, Emitech, USA) for visualization under the scanning electron microscope (SEM) (SU‐70, Hitachi, Japan) and energy dispersive X‐ray spectroscopy (EDS) (QUANTAX200 Bruker, Germany).

### Scaffold Swelling and Degradation Tests

The swelling test evaluated the capacity of absorbing water, whereas the degradation test aimed to monitor the weight loss as a function of time. NC scaffolds and controls with bare BGNP were prepared for each timepoint (0,1, 3, 7, 14, and 30 days) (*N* = 10). Initial weight (mi) was measured following scaffolds’ photocuring (day 0). The scaffolds were then immersed in Dulbecco's phosphate‐buffered saline (dPBS) and placed in an incubator at 37 °C. At each time point, the scaffolds’ weight (wet weight (mw)) was measured after excess water was removed with filter paper. Afterward, the scaffolds were freeze‐dried to measure the dry weight (mf). The water uptake/swelling degree was calculated by dividing mi by the difference between mw and mi. Weight loss was calculated as the difference between mf and mi.

### Mechanical Characterization

Mechanical testing was performed using a universal mechanical testing machine (3340 series, Instron, USA) equipped with a 50 N load cell in unidirectional compressive mode. Five samples of each formulation were tested at room temperature. For the single material formulation assays, the samples were comprised of 50 µL of the formulation photocured directly in PDMS molds of 6 mm in diameter. For the case of the multi‐material and gradien constructs, each layer was comprised of 50 µL, which were pipeted to a PDMS mold with a diameter of 8 mm and photocured. Young's Modulus was calculated as the slope of the initial linear region. Toughness was measured as the area under the curve up to the break. Ultimate stress and strain were considered as the stress and strain values at the point of break, respectively. The compression assays were performed at a constant compression rate of 1 mm min^−1^. For the stress relaxation assays, the strain was set to either 33 or 50% with 10 mN of pre‐load.

### Cell Culture, Seeding, and Bioink Encapsulation

The biological performance of BSAMA‐ and HAMA‐based NC inks was evaluated using in‐house isolated primary human adipose stem cells (hASC) at passage 5. The cells were cultured in α‐MEM (Thermo Fisher Scientific, USA) supplemented with 10% v/v of fetal bovine serum (Thermo Fisher Scientific, USA) and 1% v/v of antibiotics (Thermo Fisher Scientific, USA), and the medium was changed every 2 days. The cells and experiments were maintained in an incubator with a 5% CO_2_ atmosphere at 37 °C. Seeding of the hydrogels was performed with cells that reached 80% sub‐confluence. For these experiments, sterile, filtered starting solutions of BSAMA, HAMA, LAP, EDC, and NHS were used, as well as UV‐sterilized BGNP. The NC ink production was performed in a Class II biological safety cabinet to maintain sterility. The cylindrical constructs were produced by cutting and retrieving them from a surrogate printed and photocured scaffold composed of intercalated HAMA and BSAMA NC inks. These constructs were then directly placed in non‐adherent 96‐well plates. The cell suspension was produced by trypsinization (trypsin/EDTA solution, Sigma‐Aldrich, Germany), and 1 × 10^5^ cells, dispersed in 200 µL of α‐MEM, were deposited on top of each scaffold. To ensure homogeneous seeding of the constructs, the plates were left in a microplate shaker (IKA, Germany) overnight. The constructs were maintained in culture for 7 days.

### Metabolic Activity

The printed multi‐material constructs and bulk HAMA and BSAMA NC constructs’ cytotoxicity and cellular proliferation were evaluated with the AlamarBlue cell viability assay. Briefly, the constructs were washed in dPBS for 5 min and then placed in a 10% v/v solution of AlamarBlue in α‐MEM. The constructs were then incubated for 5 h at 37 °C, protected from light. The fluorescence was quantified in triplicate in black opaque plates at an excitation of 540 and 600 nm emission in a microplate reader (Synergy HTX, Biotek, USA).

### Live/Dead Assay

To qualitatively assess the cell proliferation in the multi‐material constructs, a calcein and propidium iodine staining was performed for microscopical evaluation. Briefly, the constructs were washed in dPBS for 5 min and incubated in a dPBS solution with 2 µL mL^−1^ of calcein AM and 1 µL mL^−1^ of propidium iodide (Thermo Fisher Scientific, USA) for 30 min. at 37 °C. Afterward, the constructs were washed thrice in dPBS and evaluated in a fluorescence microscope (Zeiss Axio Imager M2, Zeiss, Germany).

### Cell Morphology Staining

The morphology of the hASCs seeded on the multi‐material constructs was evaluated by DAPI/phalloidin staining. The constructs were washed in dPBS for 5 min and fixed using a 4% paraformaldehyde solution. Afterward, the constructs were incubated with a DAPI solution (1 µL in 1000 µL of dPBS) for 30 min. The constructs were then washed twice and incubated with a phalloidin solution (5 µL in 1000 µL of dPBS) for 45 min. Finally, the constructs were washed thrice and observed under a fluorescence microscope (Zeiss Axio Imager M2, Zeiss, Germany).

### Finite Element Simulations

Based on experimental results, an isotropic visco‐hyperelastic material response was adopted. The hyperelastic behavior was modeled using the Yeoh formulation,^[^ ^88^ ^]^ while the viscous effects were captured through the generalized Maxwell model.^[^ ^89^, ^90^ ^]^ This constitutive model was implemented in the ABAQUS finite element software using a user‐defined UMAT subroutine, as previously described elsewhere.^[^ ^89^, ^90^ ^]^

A micro‐genetic algorithm was developed in MATLAB and integrated with Python and ABAQUS to calibrate the model parameters. The algorithm began by generating a random population of five individuals, each representing a unique set of material parameters. The fitness of each individual was evaluated through a defined objective function, and the fittest individual was directly passed on to the next generation. The remaining individuals underwent a tournament selection process, where the winners serve as “parents” for the next generation, produced via a crossover strategy. To maintain genetic diversity, the population was checked at each generation. If diversity was lacking, the population was reinitialized randomly, preserving only the fittest individual. This iterative process continued until the algorithm converged. To provide experimental validation, cylindrical finite element models were developed using hybrid C3D8 elements. The models were subjected to compression followed by hold steps to evaluate the viscous response, as evidenced by stress relaxation behavior. The numerical simulation replicated the experimental setup to ensure more reliable and consistent results for comparison between the two approaches. For the clinical scenario of applying mechanical forces involved in mandibular joint restoration, the numerical model of the mandible was developed by Charité Universitätsmedizin in Berlin; where load, boundary, and contact conditions, as well as mechanical parameters, can be found elsewhere.^[^ ^85^ ^]^

### Statistical Analysis

Data were presented as mean ± standard deviation for the experiments with at least three independent assays. Statistical analyses were performed with GraphPad Prism 10 (GraphPad Software, California, USA) using one‐way and two‐way analysis of variance (ANOVA) and the Tukey test for post hoc assessments of the differences between samples. Statistical significance was defined as *p < 0.05, **p < 0.01, ***p < 0.001, ****p < 0.0001, ns = not significant.

## Conflict of Interest

The authors declare no conflict of interest.

## Supporting information



Supporting Information

## Data Availability

The data that support the findings of this study are available from the corresponding author upon reasonable request.
