# Supplementary material for: Natural‐Based Nanocomposite Ink Engineering for Seamless Multi‐Material Integration in Extrusion‐Based 3D Printing
Source: Adv Healthc Mater. 2025 Sep 6;15(1):e02733. doi: 10.1002/adhm.202502733 (PMC12790321; doi:10.1002/adhm.202502733)
Supplement: Supplementary file 1 — Supporting Information [file ADHM-15-0-s001.pdf]

ADVANCED HEALTHCARE MATERIALS

Supporting Information

for *Adv. Healthcare Mater.*, DOI 10.1002/adhm.202502733

Natural-Based Nanocomposite Ink Engineering for Seamless Multi-Material Integration in Extrusion-Based 3D Printing

João R. Maia, Miguel Bilo, Daniel S. Fidalgo, Pedro D. Rebolo, Ana S. Silva, Marco Parente, Rita Sobreiro-Almeida and João F. Mano**

Natural-based nanocomposite ink engineering for seamless multi-material integration in extrusion-based 3D printing

João R. Maia[1], Miguel Bilo[1], Daniel S. Fidalgo[2], Pedro D. Rebolo[2], Ana S. Silva[1], Marco Parente[2], Rita Sobreiro-Almeida[1*], João F. Mano[1*]

[1] Department of Chemistry, CICECO – Aveiro Institute of Materials, University of Aveiro, 3810-193, Aveiro, Portugal

[2] Institute of Science and Innovation in Mechanical and Industrial Engineering (INEGI), R. Dr. Roberto Frias 400, 4200-465, Porto, Portugal

[*] Corresponding authors: rita.almeid@ua.pt, jmano@ua.pt

Supplementary information

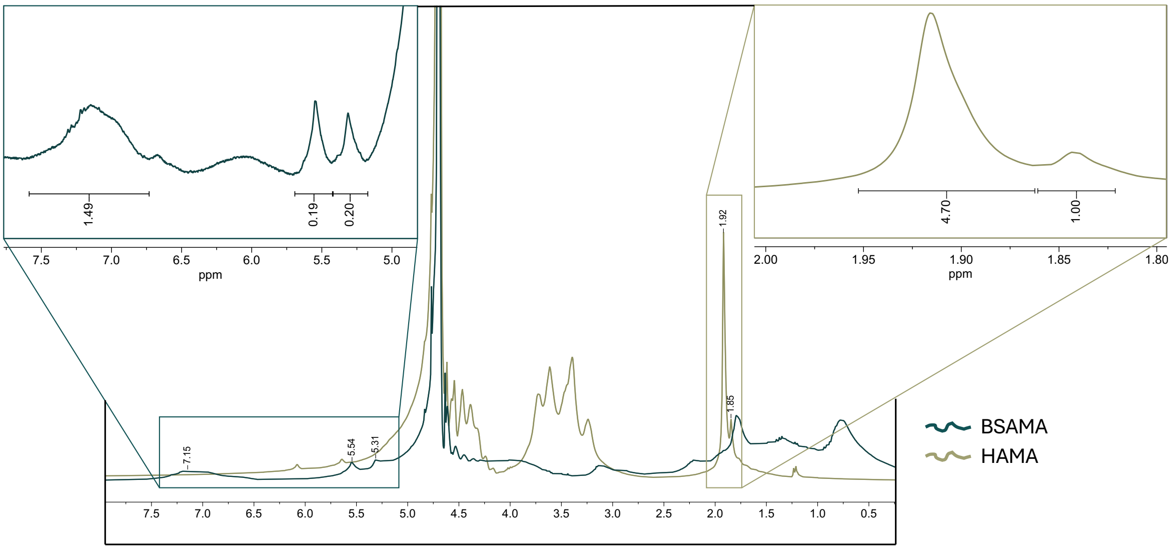

Figure S1-^{1}H-NMR of HAMA and BSAMA with highlighted peaks used to calculate respective DOM.

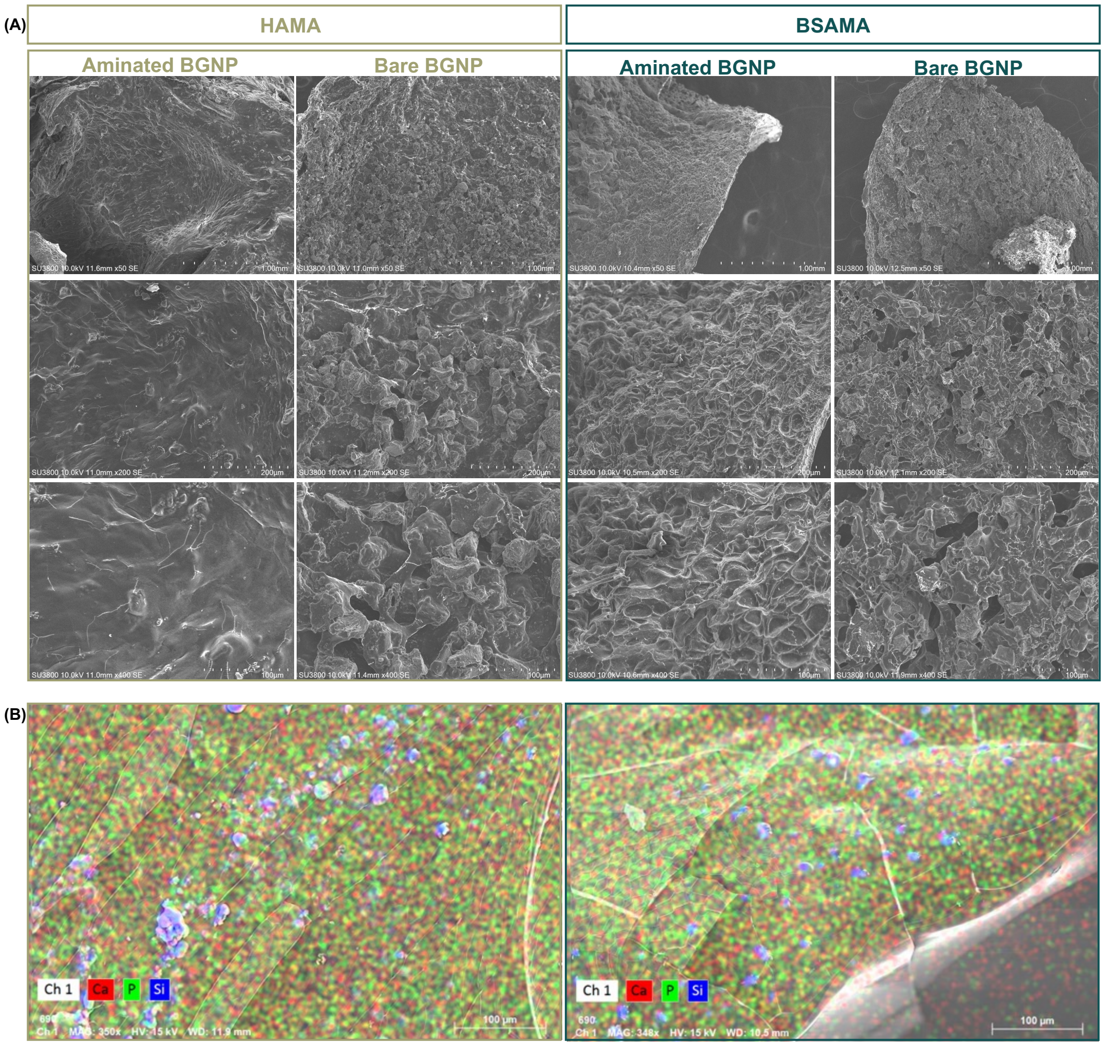

Figure S2- (A) SEM images of HAMA and BSAMA NC scaffolds with 10 % aminated and bare BGNP after photocrosslinking. (B) SEM-EDS images of HAMA and BSAMA NC scaffolds with 10 % BGNP (before SBF immersion).

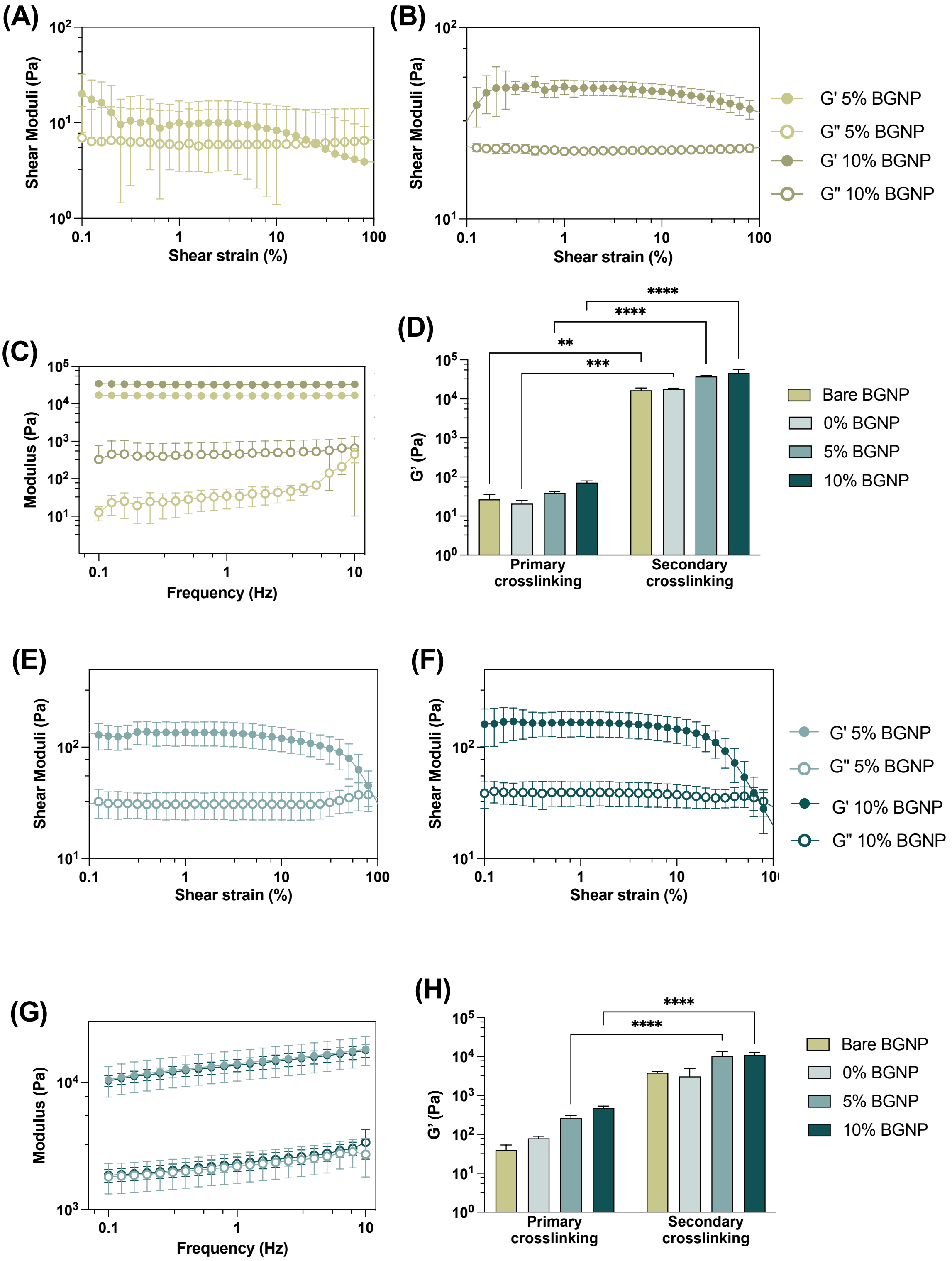

Figure S3-(A) Shear strain sweep of HAMA NC inks with 5 % BGNP. (B) Shear strain sweep of HAMA NC inks with 10 % BGNP. (C) Frequency sweep of HAMA NC inks with 5 and 10 % BGNP. (D) Average storage moduli after the primary and secondary crosslinking for HAMA NC inks and controls. (E) Shear strain sweep of BSAMA NC inks with 5 % BGNP. (F) Shear strain sweep of BSAMA NC inks with 10 % BGNP. (G) Frequency sweep of BSAMA NC inks with 5 and 10 % BGNP. (H) Average storage moduli after the primary and secondary crosslinking for BSAMA NC inks and controls.

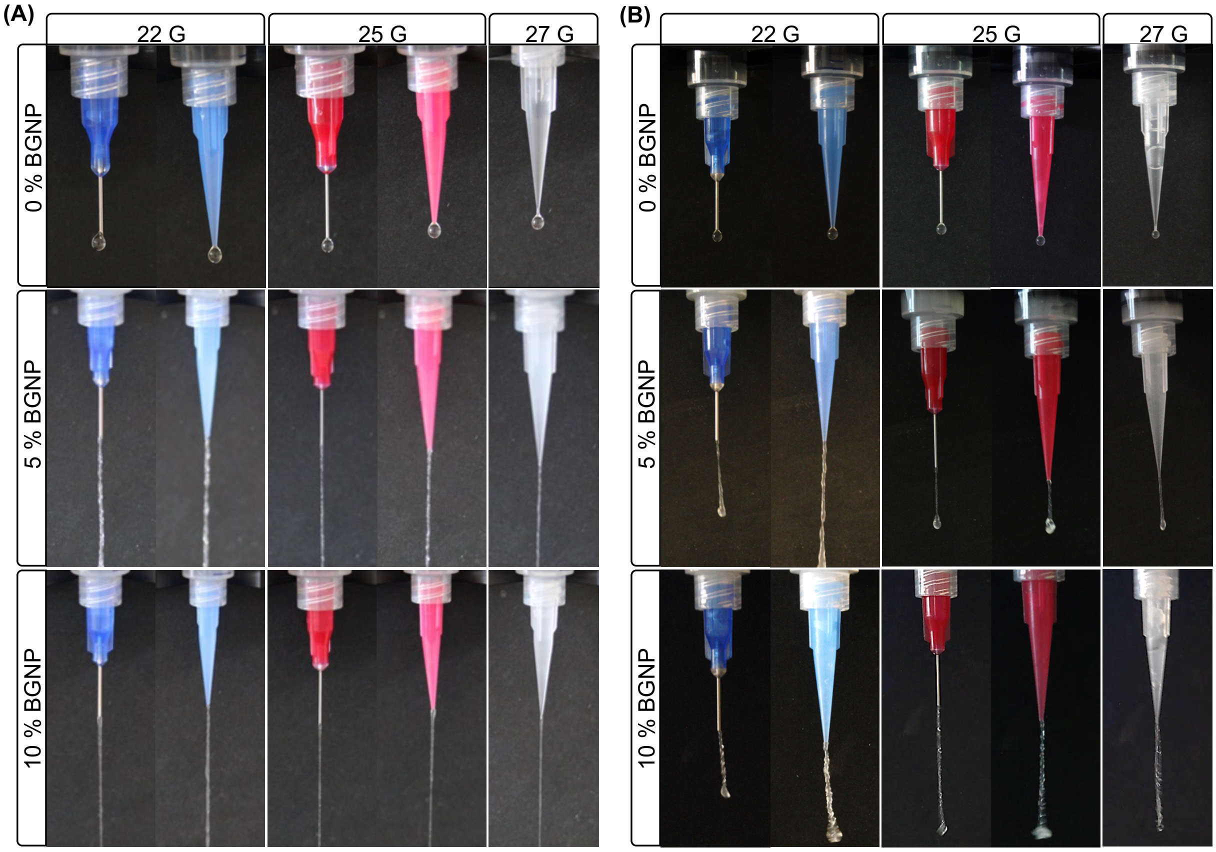

Figure S4- Ink extrudability testing of NC inks with 0, 5, and 10 % BGNP through different gauge needles and nozzles using a pump, where (A) depicts HAMA NC inks and (B) depicts BSAMA NC inks.

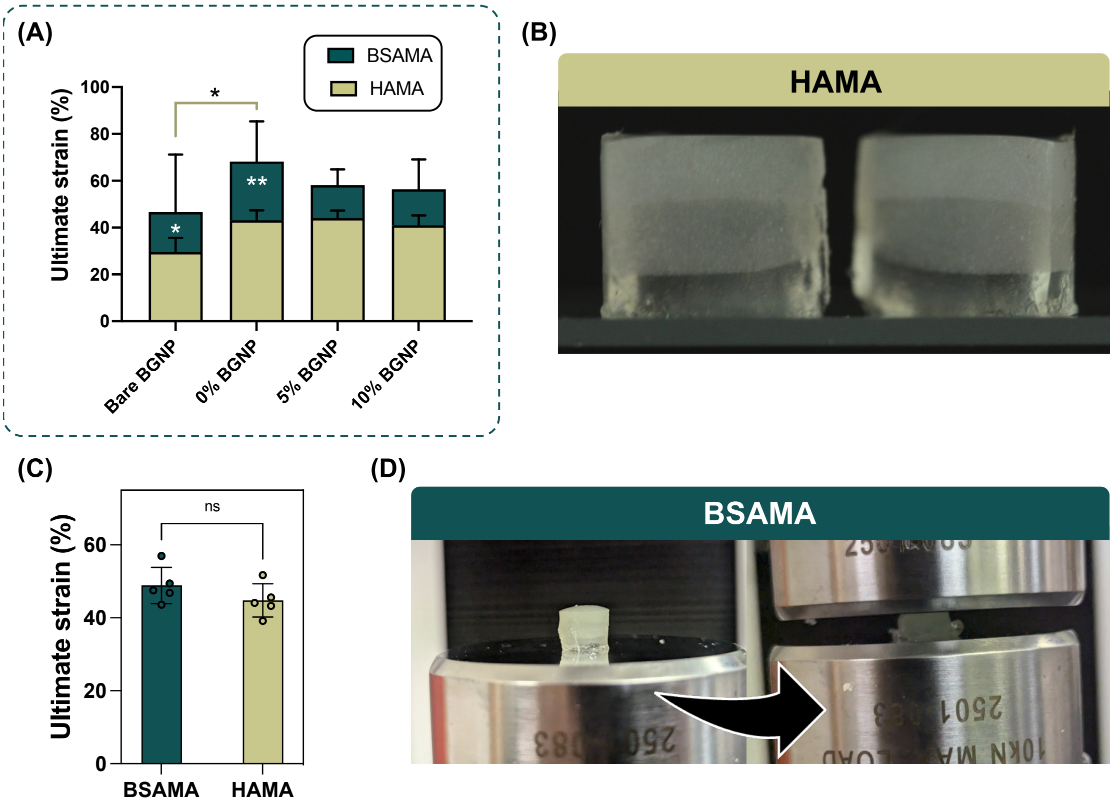

Figure S5-(A) Ultimate strain of BSAMA and HAMA NC scaffolds of 0, 5, and 10 % of BGNP of 10 % of bare BGNP. (B) HAMA-based BGNP gradient constructs composed of layers of 0, 5, and 10 % of BGNP cut in half. (C) Ultimate strain of BSAMA and HAMA-based BGNP gradient constructs. (D) BSAMA-based BGNP gradient constructs compression mechanical testing.

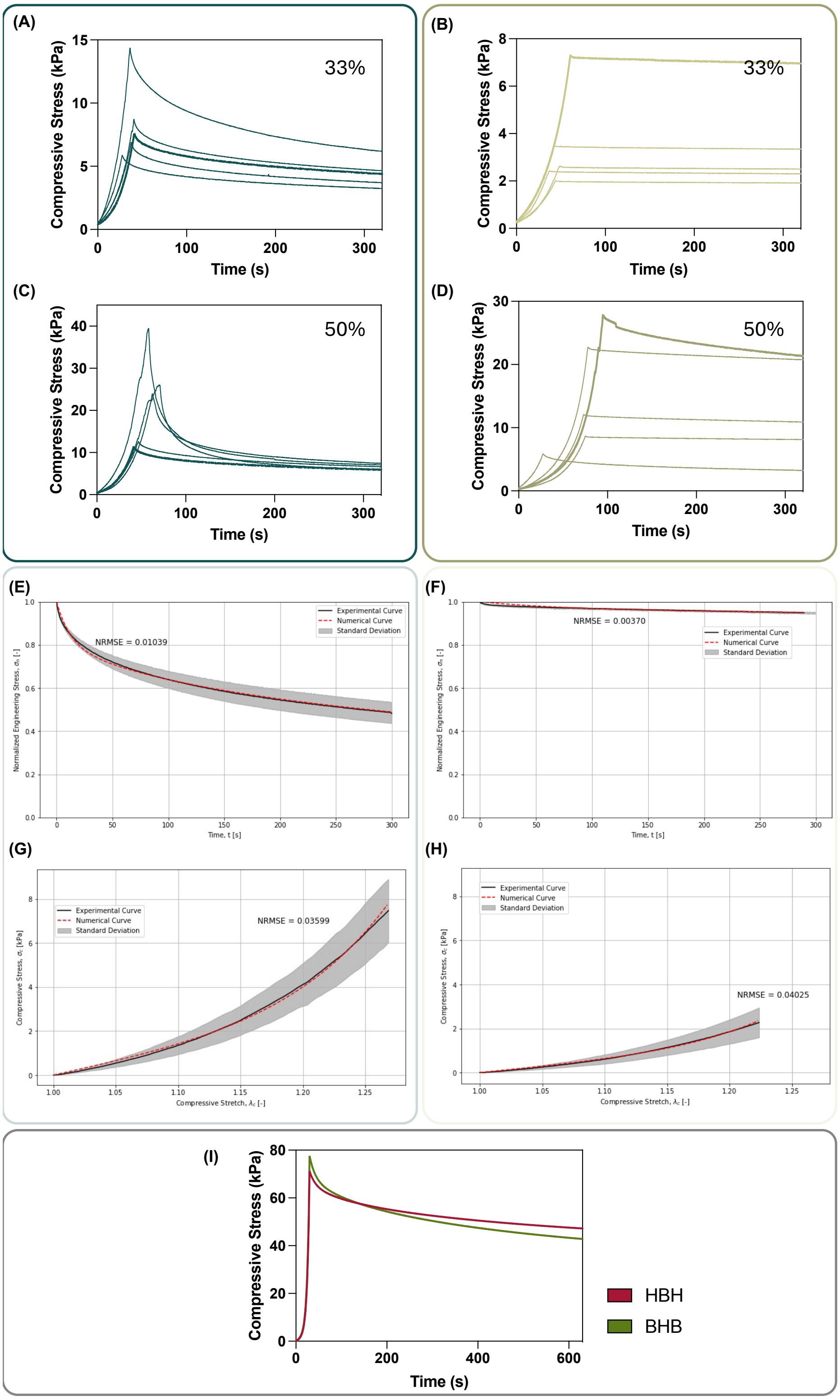

Figure S6- (A) stress-relaxation curves at 33 % strain for BSAMA 5 % BGNP NC. (B) stress-relaxation curves at 33 % strain for HAMA 5 % BGNP NC. (C) stress-relaxation curves at 50 % strain for BSAMA 5 % BGNP NC. (D) stress-relaxation curves at 50 % strain for HAMA 5 % BGNP NC. (E) and (F) Fitting of the optimized numerical curve from the compression step to the respective experimental curve for 5 % BGNP BSAMA and HAMA NC, respectively. (G) and (H) Fitting of the optimized numerical curve from the relaxation step to the respective experimental curve for 5 % BGNP BSAMA and HAMA NC, respectively. (I) Computer-simulated stress-relaxation curve at 50 % strain of HBH and BHB constructs.

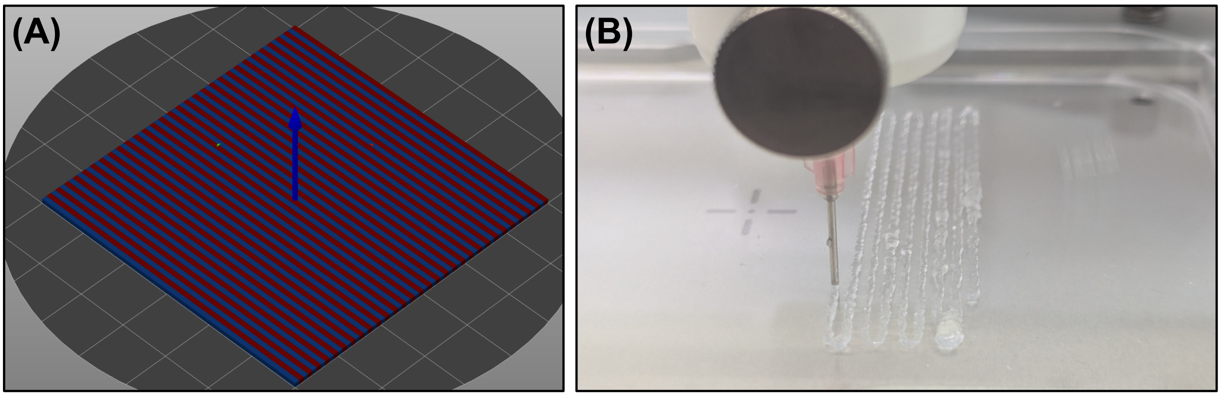

Figure S7- (A) G-code of the 6 by 6 cm structure composed of interfaced 1mm filaments of BSAMA and HAMA NC inks represented in blue and red to be printed simultaneously. (B) Printing of the BSAMA NC ink.

Table S1 – Optimized mechanical properties of (A) 5 % BGNP BSAMA NC and (B) 5 % BGNP HAMA NC.

(A)

Hyperelastic Behavior	Viscous Behavior
$C_{10} = 5.900e - 4$ MPa	$\alpha = 3$ (-)
$C_{20} = 9.600e - 4$ MPa	$\beta_1 = 0.72$ (-)
$C_{30} = 9.300e - 4$ MPa	$\tau_1 = 12$ s
	$\beta_2 = 0.41$ (-)
	$\tau_2 = 76$ s
	$\beta_3 = 1.5$ (-)
	$\tau_3 = 429$ s

(B)

Hyperelastic Behavior	Viscous Behavior
$C_{10} = 8.440e - 4$ MPa	$\alpha = 3$ (-)
$C_{20} = 1.334e - 3$ MPa	$\beta_1 = 0.00$ (-)
$C_{30} = 2.254e - 3$ MPa	$\tau_1 = 39$ s
	$\beta_2 = 0.04$ (-)
	$\tau_2 = 76$ s
	$\beta_3 = 0.09$ (-)
	$\tau_3 = 1343$ s
